# Biodiversity of *Lecanosticta* pine-needle blight pathogens suggests a Mesoamerican Centre of origin

**DOI:** 10.1186/s43008-019-0004-8

**Published:** 2019-06-07

**Authors:** Ariska van der Nest, Michael J. Wingfield, Paulo C. Ortiz, Irene Barnes

**Affiliations:** 1 Forestry and Agricultural Biotechnology Institute (FABI), Department of Biochemistry, Genetics and Microbiology, University of Pretoria, Pretoria, 0002 South Africa; 2 Instituto Nacional de Bosques (INAB), Guatemala City, Guatemala

**Keywords:** Brown spot needle blight, *Lecanosticta*, Mesoamerica, *Pinus* pathogens, phylogeny

## Abstract

*Lecanosticta acicola* causes the disease known as brown spot needle blight (BSNB), on *Pinus* species. The pathogen is thought to have a Central American centre of origin. This was based on the morphological variation between isolates believed to represent *L. acicola* from native *Pinus* spp. Two species of *Lecanosticta*, *L. brevispora* and *L. guatemalensis*, have recently been described from Mexico and Guatemala respectively based on morphology and sequence-derived phylogenetic inference. However, the putative native pathogen, *L. acicola,* was not found in those areas. In this study, the species diversity of a large collection of *Lecanosticta* isolates from Central America was considered. Phylogenetic analyses of the *BT*1, ITS, *MS204*, *RPB*2 and *TEF*1 gene regions revealed six species of *Lecanosticta*, four of which represented undescribed taxa. These are described here as *Lecanosticta jani* sp. nov. from Guatemala and Nicaragua, *L. pharomachri* sp. nov. from Guatemala and Honduras, *L. tecunumanii* sp. nov. from Guatemala and *L. variabilis* sp. nov. from Guatemala, Honduras, and Mexico. New host and country records were also found for the previously described *L. brevispora* and *L. guatemalensis. Lecanosticta acicola* was not found in any of the samples from Central America, and we hypothesize that it could be a northern hemisphere taxon. The high species diversity of *Lecanosticta* found in Mesoamerica suggests that this is a centre of diversity for the genus.

**Electronic supplementary material:**

The online version of this article (10.1186/s43008-019-0004-8) contains supplementary material, which is available to authorized users.

## INTRODUCTION

Brown spot needle blight (BSNB) or Lecanosticta needle blight is an important needle disease on *Pinus* species. The disease is characterised by brown spots on necrotic yellow lesions at the points of infection and die-back of the needles from the apex, which often leads to premature defoliation (Ivory 1987). BSNB is caused by the fungal pathogen, *Lecanosticta acicola* (Siggers 1944). The fungus is a well-known pathogen in the USA and has also been recorded in Central America, Colombia, Europe as well as Asian countries including China, Japan and Korea. *Lecanosticta acicola* is regarded as an A2 quarantine pathogen in Europe and Colombia where it is present as well as an A1 quarantine pathogen in the rest of South America (COSAVE), Africa (IASPC) and the Eurasian Economic Union countries where it has yet to be recorded (https://gd.eppo.int/taxon/SCIRAC/categorization). Despite its quarantine status, *L. acicola* has been discovered in various new locations and on new hosts in Europe during the past decade (Jankovsky et al. 2009; Markovskaja et al. 2011; Anonymous 2012; Hintsteiner et al. 2012; Adamson et al. 2015; Janoušek et al. 2016; Ortíz de Urbina et al. 2017; Mullett et al. 2018; Cleary et al. 2019; Sadiković et al. 2019).

Siggers (1944) and Evans (1984) summarised the taxonomic and nomenclatural history of *Lecanosticta acicola*, which was complicated by the former system which allowed asexual and sexual morphs of the same species of fungi to be given separate scientific names (Kais 1971; Evans 1984). From 1972 to 2012, the name *Mycosphaerella dearnessii* was widely used for the causal agent of BSNB. It was, however, recently recognised that *Mycosphaerella* is polyphyletic and should be strictly used for fungi in *Ramularia* (Crous et al. 2007; Crous 2009). Following the One Fungus One Name (1F1N) convention (Hawksworth et al. 2011), the nomenclatural rules were changed in July 2011, and included in subsequent editions of the *International Code of Nomenclature for algae, fungi, and plants* (ICN) (Turland et al. 2018). *Lecanosticta* was taken up as the appropriate name, with *L. acicola* as type species of the genus (Crous et al. 2009a; Quaedvlieg et al. 2012).

Five species of *Lecanosticta* have been described: *Lecanosticta acicola, L. brevispora*, *L. guatemalensis* (Quaedvlieg et al. 2012), *L. gloeospora* (Evans 1984), and *L. longispora* (Marmolejo 2000)*. Lecanosticta acicola* remains the best-known species and records suggest that it has a wide distribution in North and South America, Europe, and Asia (https://gd.eppo.int/taxon/SCIRAC/distribution). The remaining four species are known only from Mesoamerica (Evans 1984; Marmolejo 2000; Quaedvlieg et al. 2012). *Lecanosticta gloeospora* was described, based only on morphology, from disease symptoms on *Pinus pseudostrobus* from Iturbide, Nuevo León, Mexico (Evans 1984). It was subsequently reported on *P. pseudostrobus* collected in 1990 in Mexico (Marmolejo 2000). *Lecanosticta longispora* was originally described from *Pinus culminicola* in Nuevo León, Mexico, based on morphology (Marmolejo 2000). Quaedvlieg et al. (2012) redescribed and epitipified *L. longispora* based on DNA sequence and morphological data. Quaedvlieg et al. (2012) delineated *Mycosphaerella* species of quarantine significance in Europe, including isolates believed to be *L. acicola* from Central America. Those isolates were distinct taxa and were named *L. brevispora* and *L. guatemalensis* from *Pinus* sp. in Mexico and from *P. oocarpa* in Guatemala.

Names assigned to *Lecanosticta* species prior to 2012 were based only on morphological characteristics. Cryptic diversity in *Lecanosticta* is illustrated by *L. guatemalensis* (IMI281598), which was initially identified as *L. acicola* (Evans 1984; Quaedvlieg et al. 2012). Identifications made utilising only morphological characteristics should clearly be re-evaluated using DNA sequence data and phylogenetic inference.

Central America is believed to be the centre of origin of *L. acicola*. This hypothesis was first proposed by Evans (1984), when the fungus was isolated from native trees in pristine forests. In a recent phylogenetic study, high levels of diversity were found in the Translation Elongation 1-α gene region (*TEF*1) of isolates from Mexico and Guatemala (Janoušek et al. 2016). Furthermore, Central American isolates did not group in the same clade as isolates from Asia, Europe, and North America. Likewise, Janoušek et al. (2016) reported poor amplification of microsatellite regions that had been developed for *L. acicola* suggesting that the isolates could represent cryptic species. The present study emerged from an opportunity to collect pine needles infected with *Lecanosticta* spp. in Guatemala, Honduras and Nicaragua from 2010 to 2012. Specimens were identified based on DNA sequence comparisons and an attempt was made to confirm whether *L. acicola* occurs in Central America.

## MATERIALS AND METHODS

### Collections used in the study

Specimens prepared from ex-type cultures and other representatives of all known *Lecanosticta* species and closely related species (Quaedvlieg et al. 2012) were obtained from the culture collection of the Westerdijk Fungal Biodiversity Institute, Utrecht, The Netherlands (CBS), and from the UK National Fungus Collection maintained by CABI Bioscience (Egham, UK: IMI). Living cultures or DNA of six isolates from Central America examined by Evans (1984), and believed to represent *L. acicola,* were also acquired from IMI (Table 1). Furthermore, isolates of *Dothistroma septosporum, D. pini, Phaeophleospora eugenia, P. gregaria,* and *Amycosphaerella africana* that represent genera in *Mycosphaerellaceae* closely related to *Lecanosticta* (Quaedvlieg et al. 2012) were included for comparative purposes. These cultures were obtained from CBS and the culture collection (CMW) of the Forestry and Agricultural Biotechnology Institute (FABI) in Pretoria, South Africa (Table 1).

**Table 1:** Details of isolates used in this study

Species	CMW number^a^	Other collection number^b^	Sampling site (Country, Region, Location)	Host	Collection date	Collector	GenBank accession numbers^d^				
							ITS	*TEF1*	*BT1*	*MS204*	*RPB2*
*Amycosphaerella africana*	45395	CBS 110843	South Africa, Western Cape Province, Pampoenvlei	*Eucalyptus cladocalyx*	Nov 1994	Crous PW	KF901702	JX901653	MK015047	MK015515	MK015290
*A. africana*	45396	CBS 680.95	South Africa, Western Cape Province, Stellenbosch mountain	*E. viminalis*	Oct 1994	Crous PW	AY626981	KF903117	MK015048	MK015516	MK015291
*Dothistroma pini*	10930	CBS 116485	USA, Michigan, Montcalm County, Crystal Lake	*Pinus nigra*	2001	Adams G, Barnes I	AY808301	AY808266	AY808196	NA	MK015292
***D. pini***	**10951**	**CBS 116487**	**USA, Michigan, Montcalm County, Stanton**	***P. nigra***	**2001**	**Adams G, Barnes I**	**AY808302**	**AY808267**	**AY808197**	**NA**	**MK015293**
***D. septosporum***	**44656**	**CBS 140339**	**Russia, St. Petersburg, Park Sosnovka**	***P. sylvestris***	**Nov 2013**	**Drenkhan R, Musolin D, Adamson K**	**KU948400**	**MK015397**	**MK015049**	**MK015517**	**MK015294**
*D. septosporum*	44657	CBS 141531	Russia, St. Petersburg, Park Sosnovka	*P. sylvestris*	Nov 2013	Drenkhan R, Musolin D, Adamson K	KU948401	MK015398	MK015050	MK015518	MK015295
*Lecanosticta acicola*	9985	CBS 871.95	France	*P. radiata*	Apr 1995	Morelet M	GU214663	MK015399	MK015051	MK015519	MK015296
*L. acicola*	45426	CBS133790	Lithuania	*P. mugo*	2009	Markovskaja S, Kacergius A, Treigiene A	HM367708	JX901645	MK015052	MK015520	MK015297
***L. acicola***	**45427**	**CBS 133791**	**USA, New Hampshire, Blackwater**	***P. strobus***	**Jun 2011**	**Ostrofsky B**	**KC012999**	**KC013002**	**MK015053**	**MK015521**	**MK015298**
*L. acicola*	45428	CBS 322.33	USA	*P. palustris*	Feb 1933	Siggers PV	MK015156	MK015400	MK015054	MK015522	MK015299
*L. acicola*	50541		Lithuania, Curonian Spit, Juodkrante	*P. mugo*	Sep 2014	Markovskaja S	MK015157	MK015401	MK015055	MK015523	MK015300
*L. acicola*	50542		Lithuania, Curonian Spit, Juodkrante	*P. mugo*	Sep 2014	Markovskaja S	MK015158	MK015402	MK015056	MK015524	MK015301
*L. brevispora*	^- e^	1A.N5S2	Guatemala, Chimaltenango, Tecpán, Finca La Esperanza	*P. pseudostrobus*	Jun 2011	Barnes I	MK015159	MK015403	–	–	–
*L. brevispora*	^- e^	1C.N1S3	Guatemala, Chimaltenango, Tecpán, Finca La Esperanza	*P. pseudostrobus*	Jun 2011	Barnes I	MK015160	MK015404	MK015057	NA	NA
*L. brevispora*	^- e^	1C.N5S4	Guatemala, Chimaltenango, Tecpán, Finca La Esperanza	*P. pseudostrobus*	Jun 2011	Barnes I	MK015161	MK015405	MK015058	MK015525	MK015302
*L. brevispora*	^- e^	1C.N6S2	Guatemala, Chimaltenango, Tecpán, Finca La Esperanza	*P. pseudostrobus*	Jun 2011	Barnes I	MK015162	MK015406	–	–	–
*L. brevispora*	^- e^	1D.N1S3	Guatemala, Chimaltenango, Tecpán, Finca La Esperanza	*P. pseudostrobus*	Jun 2011	Barnes I	MK015163	MK015407	MK015059	NA	NA
*L. brevispora*	^- e^	IB31.4a	Guatemala, Alta Verapaz, Santa Cruz Verapaz, near Tactíc	*P. oocarpa*	Oct 2010	Barnes I	MK015164	MK015408	MK015060	MK015526	MK015303
*L. brevispora*	36894		Guatemala, Finca La Soledad (near Jalapa), Mataquescuintla	*P. pseudostrobus*	Oct 2010	Barnes I	MK015165	MK015409	MK015061	NA	MK015304
*L. brevispora*	37123		Guatemala, Alta Verapaz, Santa Cruz Verapaz, near Tactíc	*P. oocarpa*	Oct 2010	Barnes I	MK015166	MK015410	NA	NA	MK015305
*L. brevispora*	42646		Honduras	*P. oocarpa*	–	–	MK015167	MK015411	MK015062	MK015527	MK015306
*L. brevispora*	42647		Guatemala, Lugar, La Soledad, Jalapa	*P. oocarpa*	Oct 2010	Barnes I	MK015168	MK015412	MK015063	MK015528	MK015307
***L. brevispora***	**45424**	**CBS 133601**	**Mexico**	***Pinus*** **sp.**	**Oct 2009**	**Yanes-Morales M**	**JX901763**	**JX901649**	**MK015064**	**MK015529**	**MK015308**
*L. brevispora*	46499		Guatemala, Chimaltenango, Tecpán, Finca La Esperanza	*P. pseudostrobus*	Jun 2011	Barnes I	MK015169	MK015413	–	–	–
*L. brevispora*	46500		Guatemala, Chimaltenango, Tecpán, Finca La Esperanza	*P. pseudostrobus*	Jun 2011	Barnes I	MK015170	MK015414	–	–	–
*L. brevispora*	46501		Guatemala, Chimaltenango, Tecpán, Finca La Esperanza	*P. pseudostrobus*	Jun 2011	Barnes I	MK015171	MK015415	MK015065	NA	NA
*L. brevispora*	46502		Guatemala, Chimaltenango, Tecpán, Finca La Esperanza	*P. pseudostrobus*	Jun 2011	Barnes I	MK015172	MK015416	–	–	–
*L. brevispora*	46503		Guatemala, Chimaltenango, Tecpán, Finca La Esperanza	*P. pseudostrobus*	Jun 2011	Barnes I	MK015173	MK015417	MK015066	MK015530	MK015309
*L. brevispora*	46504		Guatemala, Chimaltenango, Tecpán, Finca La Esperanza	*P. pseudostrobus*	Jun 2011	Barnes I	MK015174	MK015418	MK015067	MK015531	MK015310
*L. brevispora*	46505		Guatemala, Chimaltenango, Tecpán, Finca La Esperanza	*P. pseudostrobus*	Jun 2011	Barnes I	MK015175	MK015419	NA	NA	MK015311
*L. brevispora*	46506		Guatemala, Chimaltenango, Tecpán, Finca La Esperanza	*P. pseudostrobus*	Jun 2011	Barnes I	MK015176	MK015420	–	–	–
*L. brevispora*	46507		Guatemala, Chimaltenango, Tecpán, Finca La Esperanza	*P. pseudostrobus*	Jun 2011	Barnes I	MK015177	MK015421	–	–	–
*L. brevispora*	46508		Guatemala, Chimaltenango, Tecpán, Finca La Esperanza	*P. pseudostrobus*	Jun 2011	Barnes I	MK015178	MK015422	–	–	–
*L. brevispora*	46509		Guatemala, Chimaltenango, Tecpán, Finca La Esperanza	*P. pseudostrobus*	Jun 2011	Barnes I	MK015179	MK015423	–	–	–
*L. brevispora*	46510		Guatemala, Chimaltenango, Tecpán, Finca La Esperanza	*P. pseudostrobus*	Jun 2011	Barnes I	MK015180	MK015424	NA	NA	MK015312
*L. brevispora*	46511		Guatemala, Chimaltenango, Tecpán, Finca La Esperanza	*P. pseudostrobus*	Jun 2011	Barnes I	MK015181	MK015425	–	–	–
*L. brevispora*	46512		Guatemala, Chimaltenango, Tecpán, Finca La Esperanza	*P. pseudostrobus*	Jun 2011	Barnes I	MK015182	MK015426	–	–	–
*L. brevispora*	46807		Guatemala, Alta Verapaz, Santa Cruz Verapaz, near Tactíc	*P. oocarpa*	Oct 2010	Barnes I	MK015183	MK015427	MK015068	MK015532	MK015313
*L. brevispora*	49291		Guatemala, Chimaltenango, Tecpán, Finca La Esperanza	*P. pseudostrobus*	Jun 2011	Barnes I	MK015184	MK015428	MK015069	NA	MK015314
*L. brevispora*	49292		Guatemala, Chimaltenango, Tecpán, Finca La Esperanza	*P. pseudostrobus*	Jun 2011	Barnes I	MK015185	MK015429	MK015070	MK015533	MK015315
*L. brevispora*	49293		Guatemala, Chimaltenango, Tecpán, Finca La Esperanza	*P. pseudostrobus*	Jun 2011	Barnes I	MK015186	NA	MK015071	MK015534	MK015316
*L. brevispora*	49294		Guatemala, Chimaltenango, Tecpán, Finca La Esperanza	*P. pseudostrobus*	Jun 2011	Barnes I	MK015187	NA	MK015072	MK015535	MK015317
*L. brevispora*	49295		Guatemala, Chimaltenango, Tecpán, Finca La Esperanza	*P. pseudostrobus*	Jun 2011	Barnes I	MK015188	NA	MK015073	MK015536	MK015318
*L. brevispora*	49296		Guatemala, Chimaltenango, Tecpán, Finca La Esperanza	*P. pseudostrobus*	Jun 2011	Barnes I	MK015189	MK015430	MK015074	MK015537	MK015319
*L. brevispora*	49297		Guatemala, Chimaltenango, Tecpán, Finca La Esperanza	*P. pseudostrobus*	Jun 2011	Barnes I	MK015190	MK015431	MK015075	MK015538	MK015320
*L. brevispora*	49298		Guatemala, Chimaltenango, Tecpán, Finca La Esperanza	*P. pseudostrobus*	Jun 2011	Barnes I	MK015191	MK015432	MK015076	MK015539	MK015321
*L. brevispora*	50523		Guatemala, Chimaltenango, Tecpán, Finca La Esperanza	*P. pseudostrobus*	Jun 2011	Barnes I	MK015192	MK015433	–	–	–
*L. brevispora*	50526		Guatemala, Chimaltenango, Tecpán, Finca La Esperanza	*P. pseudostrobus*	Jun 2011	Barnes I	MK015193	MK015434	MK015077	NA	NA
*L. brevispora*	50527		Guatemala, Chimaltenango, Tecpán, Finca La Esperanza	*P. pseudostrobus*	Jun 2011	Barnes I	MK015194	MK015435	NA	NA	MK015322
*L. brevispora*	50528		Guatemala, Chimaltenango, Tecpán, Finca La Esperanza	*P. pseudostrobus*	Jun 2011	Barnes I	MK015195	MK015436	MK015078	NA	NA
*L. brevispora*	50529		Guatemala, Chimaltenango, Tecpán, Finca La Esperanza	*P. pseudostrobus*	Jun 2011	Barnes I	MK015196	MK015437	–	–	–
*L. brevispora*	50530		Guatemala, Chimaltenango, Tecpán, Finca La Esperanza	*P. pseudostrobus*	Jun 2011	Barnes I	MK015197	MK015438	MK015079	MK015540	MK015323
*L. brevispora*	50531		Guatemala, Chimaltenango, Tecpán, Finca La Esperanza	*P. pseudostrobus*	Jun 2011	Barnes I	MK015198	MK015439	MK015080	MK015541	MK015324
*L. brevispora*	50532		Guatemala, Chimaltenango, Tecpán, Finca La Esperanza	*P. pseudostrobus*	Jun 2011	Barnes I	MK015199	MK015440	–	–	–
*L. brevispora*	51050		Guatemala, Alta Verapaz, Santa Cruz Verapaz, near Tactíc	*P. oocarpa*	Oct 2010	Barnes I	MK015200	MK015441	NA	MK015542	MK015325
***L. gloeospora*** ^**c**^	**42645**	**IMI 283812**	**Mexico, Nuevo León, Iturbide-Galeana**	***P. pseudostrobus***	**May 1983**	**Evans HC**	**KU948431**	**MK015442**	**MK015081**	**MK015543**	**MK015326**
*L. guatemalensis*	^- e^	IB30/2d	Guatemala, Alta Verapaz, Santa Cruz Verapaz, near Tactíc	*P. oocarpa*	Oct 2010	Barnes I	MK015201	MK015443	–	–	–
*L. guatemalensis*	^- e^	IB32/1a	Guatemala, Alta Verapaz, Santa Cruz Verapaz, near Tactíc	*P. oocarpa*	Oct 2010	Barnes I	MK015202	MK015444	–	–	–
*L. guatemalensis*	^- e^	IB32/2e	Guatemala, Alta Verapaz, Santa Cruz Verapaz, near Tactíc	*P. oocarpa*	Oct 2010	Barnes I	MK015203	MK015445	MK015082	NA	NA
*L. guatemalensis*	^- e^	IB35/2e	Guatemala, Chiquimula	*P. oocarpa*	Oct 2010	Barnes I	MK015204	MK015446	MK015083	NA	NA
*L. guatemalensis*	^- e^	IB35/2j	Guatemala, Chiquimula	*P. oocarpa*	Oct 2010	Barnes I	MK015205	MK015447	–	–	–
*L. guatemalensis*	^- e^	IB35/9a	Guatemala, Chiquimula	*P. oocarpa*	Oct 2010	Barnes I	MK015206	MK015448	MK015084	NA	NA
*L. guatemalensis* ^c^		IMI 275573	Honduras, Yoro	*P. oocarpa*	Oct 1980	Evans HC	MK015207	MK015449	NA	NA	NA
*L. guatemalensis* ^c^		IMI 281563	Honduras	*P. caribaea*	May 1982	Evans HC	MK015208	NA	NA	NA	NA
*L. guatemalensis* ^c^		IMI 281596	Nicaragua	*P. tecunumanii*	Nov 1981	Evans HC	MK015209	MK015450	NA	NA	NA
*L. guatemalensis*	^- e^	N3/1c	Nicaragua, Matagalpa	*P. oocarpa*	Jun 2011	Barnes I	MK015210	MK015451	MK015085	MK015544	MK015327
*L. guatemalensis*	36811		Guatemala, Jalapa, Finca Forestal Soledad	*P. maximinoi*	Oct 2010	Barnes I	MK015211	MK015452	MK015086	NA	MK015328
*L. guatemalensis*	36812		Guatemala, Coban, San Juan Chamelco	*P. maximinoi*	Oct 2010	Barnes I	MK015212	MK015453	MK015087	MK015545	MK015329
*L. guatemalensis*	37121		Guatemala, Alta Verapaz, Santa Cruz Verapaz, near Tactíc	*P. oocarpa*	Oct 2010	Barnes I	MK015213	MK015454	–	–	–
*L. guatemalensis*	37122		Guatemala, Alta Verapaz, Santa Cruz Verapaz, near Tactíc	*P. oocarpa*	Oct 2010	Barnes I	MK015214	MK015455	MK015088	MK015546	MK015330
*L. guatemalensis*	37124		Guatemala, Alta Verapaz, Santa Cruz Verapaz, near Tactíc	*P. oocarpa*	Oct 2010	Barnes I	MK015215	MK015456	–	–	–
*L. guatemalensis*	37126		Guatemala, Alta Verapaz, Santa Cruz Verapaz, near Tactíc	*P. oocarpa*	Oct 2010	Barnes I	MK015216	MK015457	MK015089	MK015547	MK015331
*L. guatemalensis*	37127		Guatemala, Alta Verapaz, Santa Cruz Verapaz, near Tactíc	*P. oocarpa*	Oct 2010	Barnes I	MK015217	MK015458	–	–	–
***L. guatemalensis*** ^**c**^	**42206**	**IMI 281598**	**Guatemala**	***P. oocarpa***	**1983**	**Evans HC**	**JX901764**	**JX901650**	**MK015090**	**MK015548**	**MK015332**
*L. guatemalensis*	43890		Guatemala, Chiquimula	*P. oocarpa*	Oct 2010	Barnes I	MK015218	MK015459	–	–	–
*L. guatemalensis*	43891		Guatemala, Chiquimula	*P. oocarpa*	Oct 2010	Barnes I	MK015219	MK015460	MK015091	NA	NA
*L. guatemalensis*	43892		Guatemala, Chiquimula	*P. oocarpa*	Oct 2010	Barnes I	MK015220	MK015461	MK015092	NA	NA
*L. guatemalensis*	43893		Guatemala, Chiquimula, San José la Arada	*P. oocarpa*	Oct 2010	Barnes I	MK015221	MK015462	MK015093	NA	NA
*L. guatemalensis*	43894		Guatemala, Chiquimula	*P. oocarpa*	Oct 2010	Barnes I	MK015222	MK015463	MK015094	NA	NA
*L. guatemalensis*	43895		Guatemala, Alta Verapaz, Santa Cruz Verapaz, near Tactíc	*P. oocarpa*	Oct 2010	Barnes I	MK015223	MK015464	MK015095	MK015549	MK015333
*L. guatemalensis*	45386		Nicaragua, Matagalpa	*P. oocarpa*	Jun 2011	Barnes I	MK015224	MK015465	–	–	–
*L. guatemalensis*	45387		Nicaragua, Matagalpa	*P. oocarpa*	Jun 2011	Barnes I	MK015225	MK015466	MK015096	MK015550	MK015334
*L. guatemalensis*	45391		Guatemala, Alta Verapaz, Santa Cruz Verapaz, near Tactíc	*P. oocarpa*	Oct 2010	Barnes I	MK015226	MK015467	MK015097	NA	NA
*L. guatemalensis*	45392		Guatemala, Alta Verapaz, Santa Cruz Verapaz, near Tactíc	*P. oocarpa*	Oct 2011	Barnes I	MK015227	MK015468	MK015098	MK015551	MK015335
*L. guatemalensis*	45393		Guatemala, Chiquimula	*P. oocarpa*	Oct 2010	Barnes I	MK015228	MK015469	–	–	–
*L. guatemalensis*	45394		Guatemala, Chiquimula	*P. oocarpa*	Oct 2010	Barnes I	MK015229	NA	–	–	–
*L. guatemalensis*	46811		Guatemala, Chiquimula	*P. oocarpa*	Oct 2010	Barnes I	MK015230	MK015470	MK015099	MK015552	MK015336
*L. guatemalensis*	46817		Guatemala, Chiquimula	*P. oocarpa*	Oct 2010	Barnes I	MK015231	MK015471	MK015100	MK015553	MK015337
*L. guatemalensis*	46819		Guatemala, Chiquimula	*P. oocarpa*	Oct 2010	Barnes I	MK015232	NA	–	–	–
*L. guatemalensis*	47108		Guatemala, Alta Verapaz, Santa Cruz Verapaz, near Tactíc	*P. oocarpa*	Oct 2010	Barnes I	MK015233	MK015472	–	–	–
*L. guatemalensis*	49400		Nicaragua, Matagalpa	*P. oocarpa*	Jun 2011	Barnes I	MK015234	MK015473	MK015101	MK015554	MK015338
*L. guatemalensis*	49402		Guatemala, Chiquimula	*P. oocarpa*	Oct 2010	Barnes I	MK015235	MK015474	MK015102	MK015555	MK015339
*L. guatemalensis*	51052		Guatemala, Chiquimula, San José la Arada	*P. oocarpa*	Oct 2010	Barnes I	MK015236	MK015475	MK015103	MK015556	MK015340
*L. guatemalensis*	51142		Nicaragua, Matagalpa	*P. oocarpa*	Jun 2011	Barnes I	MK015237	MK015476	MK015104	MK015557	MK015341
*L. jani*	^- e^	267.44.N1	Guatemala, Jalapa, Finca La Soledad, Mataquescuintla	*P. tecunumanii*	Sep 2012	Barnes I	MK015238	MK015477	MK015105	MK015558	MK015342
*L. jani*	^- e^	267.47.N1	Guatemala, Jalapa, Finca La Soledad, Mataquescuintla	*P. tecunumanii*	Sep 2012	Barnes I	MK015239	MK015478	MK015106	MK015559	MK015343
*L. jani*	^- e^	267.47.N2	Guatemala, Jalapa, Finca La Soledad, Mataquescuintla	*P. tecunumanii*	Sep 2012	Barnes I	MK015240	MK015479	MK015107	MK015560	MK015344
*L. jani*	^- e^	267.51.N2S1	Guatemala, Jalapa, Finca La Soledad, Mataquescuintla	*P. tecunumanii*	Sep 2012	Barnes I	MK015241	MK015480	NA	NA	MK015345
*L. jani*	^- e^	267.52.N1S1	Guatemala, Jalapa, Finca La Soledad, Mataquescuintla	*P. tecunumanii*	Sep 2012	Barnes I	MK015242	MK015481	MK015108	MK015561	MK015346
*L. jani*	^- e^	267.52.N2S1	Guatemala, Jalapa, Finca La Soledad, Mataquescuintla	*P. tecunumanii*	Sep 2012	Barnes I	MK015243	MK015482	MK015109	MK015562	MK015347
*L. jani*	^- e^	IB30/2b	Guatemala, Alta Verapaz, Santa Cruz Verapaz, near Tactíc	*P. oocarpa*	Oct 2010	Barnes I	MK015244	MK015483	MK015110	MK015563	NA
*L. jani*	^- e^	IB35/3c	Guatemala, Chiquimula	*P. oocarpa*	Oct 2010	Barnes I	MK015245	MK015484	MK015111	MK015564	MK015348
*L. jani*	^- e^	IB13/2f	Guatemala	*P. maximinoi*	Oct 2010	Barnes I	MK015246	MK015485	MK015112	MK015565	MK015349
*L. jani*	^- e^	N3/2c	Nicaragua, Matagalpa	*P. oocarpa*	Jun 2011	Barnes I	MK015247	NA	MK015113	MK015566	MK015350
*L. jani*	36808		Guatemala, Jalapa, Finca Forestal Soledad	*P. maximinoi*	Oct 2010	Barnes I	MK015248	NA	MK015114	MK015567	MK015351
*L. jani*	36810		Guatemala, Jalapa, Finca Forestal Soledad	*P. maximinoi*	Oct 2010	Barnes I	MK015249	NA	MK015115	MK015568	MK015352
*L. jani*	37128		Guatemala, Alta Verapaz, Santa Cruz Verapaz, near Tactíc	*P. oocarpa*	Oct 2010	Barnes I	MK015250	MK015486	MK015116	MK015569	MK015353
*L. jani*	38950	CBS 144446; PREM 62186	Guatemala, Jalapa, Finca La Soledad, Mataquescuintla	*P. oocarpa*	Sep 2012	Barnes I	MK015251	MK015487	MK015117	MK015570	MK015354
***L. jani***	**38958**	**CBS 144456; PREM 62185**	**Guatemala, Jalapa, Finca La Soledad, Mataquescuintla**	***P. oocarpa***	**Sep 2012**	**Barnes I**	**MK015252**	**MK015488**	**MK015118**	**MK015571**	**MK015355**
*L. jani*	38959		Guatemala, Jalapa, Finca La Soledad, Mataquescuintla	*P. oocarpa*	Sep 2012	Barnes I	MK015253	NA	NA	NA	NA
*L. jani*	38968		Guatemala, Jalapa, Finca La Soledad, Mataquescuintla	*P. oocarpa*	Sep 2012	Barnes I	MK015254	NA	MK015119	NA	NA
*L. jani*	45388		Guatemala	*P. maximinoi*	Oct 2010	Barnes I	MK015255	NA	MK015120	MK015573	MK015356
*L. jani*	45389		Guatemala	*P. maximinoi*	Oct 2010	Barnes I	MK015256	MK015489	MK015121	MK015574	MK015357
*L. jani*	47109		Guatemala	*P. maximinoi*	Oct 2010	Barnes I	MK015257	MK015490	MK015122	MK015575	MK015358
*L. jani*	48830		Nicaragua, Matagalpa	*P. oocarpa*	Jun 2011	Barnes I	MK015258	NA	MK015123	MK015576	MK015359
*L. jani*	48831	CBS 144447; PREM 62187	Guatemala, Alta Verapaz, Santa Cruz Verapaz, near Tactíc	*P. oocarpa*	Oct 2010	Barnes I	MK015259	MK015491	MK015124	MK015577	MK015360
*L. jani*	49401		Guatemala	*P. maximinoi*	Oct 2010	Barnes I	MK015260	MK015492	NA	MK015578	MK015361
*L. jani*	51051		Guatemala	*P. maximinoi*	Oct 2010	Barnes I	MK015261	MK015493	MK015125	MK015579	MK015362
*L. jani*	51058		Guatemala, Jalapa, Finca La Soledad, Mataquescuintla	*P. tecunumanii*	Sep 2012	Barnes I	MK015262	MK015494	MK015126	MK015580	MK015363
*L. jani*	51059		Guatemala, Jalapa, Finca La Soledad, Mataquescuintla	*P. tecunumanii*	Sep 2012	Barnes I	MK015263	MK015495	MK015127	MK015581	MK015364
*L. jani*	51143		Nicaragua, Matagalpa	*P. oocarpa*	Jun 2011	Barnes I	MK015264	NA	MK015128	MK015582	MK015365
***L. longispora***	**45429**	**CBS 133602**	**Mexico**	***Pinus*** **sp.**	**Oct 2009**	**Yanes-Morales M**	**JX901766**	**JX901651**	**MK015129**	**MK015583**	**MK015366**
*L. longispora*	45430	CPC 17941	Mexico	*Pinus* sp.	Oct 2009	Yanes-Morales M	JX901765	JX901652	MK015130	MK015584	MK015367
*L. pharomachri*	^- e^	267.8A.N2S1	Guatemala, Jalapa, Finca La Soledad, Mataquescuintla	*P. oocarpa*	Sep 2012	Barnes I	MK015265	MK015496	NA	NA	MK015368
*L. pharomachri*	^- e^	267.12.N1S2	Guatemala, Jalapa, Finca La Soledad, Mataquescuintla	*P. oocarpa*	Sep 2012	Barnes I	MK015266	NA	NA	NA	MK015369
*L. pharomachri*	^- e^	267.30.MD.N1	Guatemala, Jalapa, Finca La Soledad, Mataquescuintla	*P. oocarpa*	Sep 2012	Barnes I	MK015267	NA	NA	NA	MK015370
*L. pharomachri*	^- e^	267.30.MD.N2	Guatemala, Jalapa, Finca La Soledad, Mataquescuintla	*P. oocarpa*	Sep 2012	Barnes I	MK015268	MK015497	MK015131	NA	MK015371
*L. pharomachri*	^- e^	267.30.N4	Guatemala, Jalapa, Finca La Soledad, Mataquescuintla	*P. oocarpa*	Sep 2012	Barnes I	MK015269	MK015498	MK015132	MK015585	MK015372
*L. pharomachri*	37132		Guatemala, Baja Verapaz, San Jerónimo, Salamá	*P. tecunumanii*	Oct 2010	Barnes I	MK015270	MK015499	MK015133	MK015586	MK015373
*L. pharomachri*	37133		Guatemala, Baja Verapaz, San Jerónimo, Salamá	*P. tecunumanii*	Oct 2010	Barnes I	MK015271	MK015500	MK015134	MK015587	MK015374
*L. pharomachri*	37134		Guatemala, Baja Verapaz, San Jerónimo, Salamá	*P. tecunumanii*	Oct 2010	Barnes I	MK015272	MK015501	MK015135	MK015588	MK015375
***L. pharomachri***	**37136**	**CBS 144448; PREM 62188**	**Guatemala, Baja Verapaz, San Jerónimo, Salamá**	***P. tecunumanii***	**Oct 2010**	**Barnes I**	**MK015273**	**MK015502**	**MK015136**	**MK015589**	**MK015376**
*L. pharomachri*	38947	CBS 144695; PREM 62189	Guatemala, Jalapa, Finca La Soledad, Mataquescuintla	*P. oocarpa*	Sep 2012	Barnes I	MK015274	MK015503	MK015137	MK015590	MK015377
*L. pharomachri*	38974	CBS 144449; PREM 62190	Guatemala, Jalapa, Finca La Soledad, Mataquescuintla	*P. oocarpa*	Sep 2012	Barnes I	MK015275	MK015504	MK015138	MK015591	MK015378
*L. pharomachri*	38975		Guatemala, Jalapa, Finca La Soledad, Mataquescuintla	*P. oocarpa*	Sep 2012	Barnes I	MK015276	NA	NA	NA	NA
*L. pharomachri*	38976		Guatemala, Jalapa, Finca La Soledad, Mataquescuintla	*P. oocarpa*	Sep 2012	Barnes I	MK015277	MK015505	MK015139	NA	MK015379
*L. pharomachri*	46810		Honduras	*P. oocarpa*	–	–	MK015278	MK015506	MK015140	MK015592	MK015380
*L. pharomachri*	46813		Guatemala, Baja Verapaz, San Jerónimo, Salamá	*P. tecunumanii*	Oct 2010	Barnes I	MK015279	MK015507	MK015141	MK015593	MK015381
*L. pharomachri*	51053		Guatemala, Jalapa, Finca La Soledad, Mataquescuintla	*P. oocarpa*	Sep 2012	Barnes I	MK015280	NA	MK015142	NA	MK015382
*L. pharomachri*	51054		Guatemala, Jalapa, Finca La Soledad, Mataquescuintla	*P. oocarpa*	Sep 2012	Barnes I	MK015281	MK015508	NA	NA	MK015383
***L. tecunumanii***	**46805**	**CBS 144450; PREM 62191**	Guatemala, Baja Verapaz, San Jerónimo, Salamá	***P. tecunumanii***	**Oct 2010**	**Barnes I**	**MK015282**	**MK015509**	**MK015143**	**MK015594**	**MK015384**
*L. tecunumanii*	46812	CBS 144452; PREM 62193	Guatemala, Baja Verapaz, San Jerónimo, Salamá	*P. tecunumanii*	Oct 2010	Barnes I	MK015283	MK015510	MK015144	MK015595	MK015385
*L. tecunumanii*	49403	CBS 144451; PREM 62192	Guatemala, Baja Verapaz, San Jerónimo, Salamá	*P. tecunumanii*	Oct 2010	Barnes I	MK015284	MK015511	MK015145	MK015596	MK015386
*L. variabilis*	36809	CBS 144455; PREM 62195	Guatemala, Jalapa, Finca Forestal Soledad	*P. maximinoi*	Oct 2010	Barnes I	MK015285	MK015512	MK015146	MK015597	MK015387
*L. variabilis*	37125	CBS 144454; PREM 62194	Guatemala, Alta Verapaz, Santa Cruz Verapaz, near Tactíc	*P. oocarpa*	Oct 2010	Barnes I	MK015286	KJ938446	MK015147	MK015598	MK015388
*L. variabilis*	37129		Guatemala, Alta Verapaz, Santa Cruz Verapaz, near Tactíc	*P. oocarpa*	Oct 2010	Barnes I	MK015287	KJ938445	MK015148	MK015599	MK015389
***L. variabilis*** ^**c**^	**42205**	**IMI 281561; CBS 144453; PREM 62196**	**Honduras, Santa Barbara, Lago Yojoa**	***P. caribaea***	**Oct 1980**	**Evans HC**	**MK015288**	**MK015513**	**MK015149**	MK015600	**MK015390**
*L. variabilis*	45390		Guatemala, Alta Verapaz, Santa Cruz Verapaz, near Tactíc	*P. oocarpa*	Oct 2010	Barnes I	MK015289	MK015514	MK015150	MK015601	MK015391
*L. variabilis*	45425	CBS 133789	Mexico	*Pinus* sp.	Nov 2009	Yanez-Morales M	JX901762	JX901648	MK015151	MK015602	MK015392
*Phaeophleospora eugeniae*	45432	CPC15159	Brazil, Vicosa, Paraiso	*Eugenia uniflora*	Mar 2008	Alfenas AC	FJ493189	JX901667	MK015152	MK015603	NA
*P. eugeniae*	45433	CPC 15143	Brazil, Vicosa, Paraiso	*E. uniflora*	Mar 2008	Alfenas AC	FJ493188	JX901666	MK015153	MK015604	NA
*P. gregaria*	45434	CBS 111166	South Africa, Western Cape Province, de Hoop Nature Reserve	*Eucalyptus cladocalyx*	Sep 1995	Wood A	JX901773	JX901664	MK015154	MK015605	MK015393
*P. gregaria*	45435	CBS 114662	South Africa, Western Cape Province, Devon Valley, Stellenbosch	*Eucalyptus* sp.	Jun 1995	Crous PW	DQ302953	JX901654	MK015155	MK015606	MK015394

^a^*CMW* Culture collection of the Forestry and Agricultural Biotechnology Institute (FABI), University of Pretoria, South Africa;

^b^*CBS* Culture collection of the Westerdijk Fungal Biodiversity Institute, Utrecht, The Netherlands, *CPC* Personal collection of Pedro Crous housed at CBS, *IMI* The UK National Fungus Collection, CABI Bioscience, Egham, UK, *PREM* The dried herbarium collection of the South African National Collection of Fungi, Mycology Unit, Biosystematics Division, Plant Protection Institute, Agricultural Research Council, Pretoria, South Africa

^c^Cultures were collected by HC Evans in Central America

^d^‘-‘= was not amplified; ‘NA‘= amplification unsuccessful;

^e^ = no viable culture available that could be submitted to CMW

Ex-type isolate of each species is indicated in bold

Pine needles, showing symptoms of brown spots or bands, were collected from *Pinus* species native to Central America from 2010 to 2012 in Guatemala, as well as from Honduras and Nicaragua in 2011 (Table 1). Conidiomata formed on the needles were aseptically excised, rolled onto 2% Dothistroma Sporulating Media (DSM: 5 g yeast extract (Biolab, Merck, Modderfontein, South Africa), 20 g malt extract (Biolab) and 15 g agar (BD Difco™, Sparks, MD) per litre of distilled water) with 100 mg/L streptomycin (Sigma-Aldrich, St Louis, MO) in order to release conidia from the conidiomata as described by Barnes et al. (2004). The isolated conidiomata were incubated for one to two days at 23 °C. The plates were examined using a dissection microscope and single germinating conidia were selected and replated onto 2% DSM. The single conidial isolates were grown for 4–6 wk. on a natural day light cycle, at 23 °C.

### DNA extractions and sequencing

Fungal tissue was scraped from the surface of the cultures on 2% DSM with a sterile scalpel blade and lyophilized. The freeze-dried mycelium was homogenized using a Retsch MM301 mixer mill (Haan, Germany) and approximately 20 ng of the crushed mycelium was used as starting material for DNA extractions. DNA was extracted using a Zymo Research ZR Fungal/Bacterial DNA MiniPrep™ kit (Irvine, CA) and eluted into a final volume of 50 μl. The quality and quantity of the extracted DNA was determined using a NanoDrop ND-1000 spectrophotometer (Thermo Fischer Scientific, Waltham, MA). DNA concentrations were diluted to 20 ng/μl working stock for polymerase chain reaction (PCR) amplifications and stored at − 20 °C until further use.

The nuclear rDNA region encompassing the internal transcribed spacers (ITS) 1 and 2, along with the 5.8S rDNA region was amplified using primers ITS1 and ITS4 (White et al. 1990) and a portion of the translation elongation factor 1-α gene (*TEF*1) using primers EF1-728F (Carbone and Kohn 1999) and EF2 (O’Donnell et al. 1998) for all the isolates. The Beta-tubulin-2 gene region (*BT*2) was amplified using the primer pair T1 (O’Donnell and Cigelnik 1997) and β-Sandy-R (Stukenbrock et al. 2012) or the primers Bt2A and Bt2B (Glass and Donaldson 1995). The Beta-tubulin-1 gene region (*BT*1) was amplified using primers Bt1A and Bt1B (Glass and Donaldson 1995), the RNA polymerase II second largest subunit (*RPB*2) gene region using primers RPB2-5f2 (Sung et al. 2007) and RPB2-7cR (Liu et al. 1999) and the guanine nucleotide-binding protein subunit beta (*MS204*) using primers MS204F.cerato and MS204R.cerato (Fourie et al. 2015).

PCR reactions for each of the six regions contained 20 ng DNA, 2.5 μl 10x PCR reaction buffer, 2.5 mM MgCl_2_, 400 nM of each primer, 200 μM of each dNTP and 1 U Faststart *Taq* DNA Polymerase (Roche Diagnostics, Indianapolis, IN). Reaction volumes were adjusted to 25 μl with sterile SABAX water (Adcock Ingram, Midrand, South Africa). PCR reactions were carried out on an Applied Biosystems® Veriti® 96 well Thermal cycler (Thermo Fisher Scientific, Waltham, MA). The cycling conditions for all six gene regions included an initial denaturation step at 95 °C for 4 min, 10 cycles consisting of 94 °C for 20 s (denaturation), a 45 s annealing step according to the primer pair annealing temperature (Table 2) and an elongation step of 45 s at 72 °C. This was followed by a further 25 cycles of 94 °C for 20 s, 45 s with a 5 s extension step per cycle at the indicated annealing temperature, a 72 °C extension for 45 s and a final step of 72 °C for 10 min. The annealing temperature was set at 56 °C for ITS, 52 °C for *TEF*1, 50 °C for *BT*1, 52 °C for *BT*2, 55 °C for *MS204* and 56 °C for *RPB*2. To visualise amplified products, 5 μl PCR products were stained with 1 μl GelRed™ nucleic acid gel stain (Biotium, Fremont, CA) and separated on 2% SeaKem® LE agarose gel (Lonza, Rockland, ME) for 20 min at 100 V and viewed under a UV light using the GelDoc™ EZ Imager (BioRad, Hercules, CA). PCR products were cleaned with a 6.65% G-50 Sephadex solution (Sigma-Aldrich, St Louis, MO) following the manufacturer’s instructions using Centri-sep spin columns (Princeton Separations, Freehold, NJ).

**Table 2:** Primers used for PCR amplification and sequencing in this study

Locus	Primer name	Direction	Primer sequence 5′ to 3’	Annealing temperature used (°C)	Amplification success	Reference
*BT*1	Bt1a	Forward	TTC CCC CGT CTC CAC TTC TTC ATG	50	87.4%	Glass and Donaldson 1995
	Bt1b	Reverse	GAC GAG ATC GTT CAT GTT GAA CTC	50		Glass and Donaldson 1995
*BT*2^a^	T1	Forward	AAC ATG CGT GAG ATT GTA AGT	52	–	O’Donnell and Cigelnik 1997
	β-Sandy-R	Reverse	GCR CGN GGV ACR TAC TTG TT	52		Stukenbrock et al. 2012
	Bt2a	Forward	GGT AAC CAA ATC GGT GCT GCT TTC	52	–	Glass and Donaldson 1995
	Bt2b	Reverse	ACC CTC AGT GTA GTG ACC CTT GGC	52		Glass and Donaldson 1995
*TEF*1	EF1-728F	Forward	CAT CGA GAA GTT CGA GAA GG	52	88.2%	Carbone and Kohn 1999
	EF-2	Reverse	GGA RGT ACC AGT SAT CAT GTT	52		O’Donnell et al. 1998
ITS	ITS1	Forward	GAA GTA AAA GTC GTA ACA AGG	56	100%	White et al. 1990
	ITS4	Reverse	TCC TCC GCT TAT TGA TAT GC	56		White et al. 1990
*MS204*	MS204F.cerato	Forward	AAG GGC ACC CTC GAG GGC CAC	55	71.7%	Fourie et al. 2015
	MS204R.cerato	Reverse	GAT GGT RAC GGT GTT GAT GTA	55		Fourie et al. 2015
*RPB*2	RPB2-5f2	Forward	GGG GWG AYC AGA AGA AGG C	56	82.7%	Sung et al. 2007
	fRPB2-7cR	Reverse	CCC ATR GCT TGY TTR CCC AT	56		Liu et al. 1999

^a^*BT*2 amplification success using all primer combinations was very low and abandoned

The concentrations of the cleaned PCR products were determined using a NanoDrop ND-1000 spectrophotometer and 60–100 ng of DNA and products were sequenced in both the forward and reverse direction using the BigDye Terminator v3.1 Cycle Sequencing Kit (Thermo Fisher Scientific) on an ABI PRISM 3500xl capillary auto sequencer (Thermo Fisher Scientific).

Forward and reverse sequences were aligned and consensus sequences generated in CLC Main workbench version 8.0 (CLC Bio, https://www.qiagenbioinformatics.com/products/clc-main-workbench/). All consensus sequences generated in this study were deposited in GenBank that is hosted by the National Center for Biotechnology Information (NCBI; http://www.ncbi.nlm.nih.gov/genbank/) (Table 1).

### Data analyses

Five datasets (*BT*1, ITS, *MS204, RPB*2 and *TEF*1) were generated and analysed individually. A partition homogeneity test (PHT) was performed with the software package PAUP* 4.0b10 (Swofford 2003) to test congruence between the five gene regions and a sixth dataset, where sequences were available for all five gene regions, was compiled and analysed. The *BT*1, ITS, *MS204* and *RPB*2 datasets included all of the sequences generated in this study and additional sequences available from GenBank (Table 1). The *TEF*1 dataset included all of the sequence data generated in this study as well as additional sequences representing 14 different *TEF*1 haplotypes of *L. acicola* (including possible cryptic species) (Janoušek et al. 2016) that were downloaded from GenBank (Table 3). Sequences for all datasets were aligned with the online version of MAFFT Version 7 (Katoh and Standley 2013; http://mafft.cbrc.jp/alignment/server/) using default settings. Aligned data were imported into MEGA 7.0.14 (Kumar et al. 2016) and manually checked and adjusted.

**Table 3:** GenBank numbers of *Lecanosticta acicola TEF*1 haplotypes included in the *TEF*1 phylogenetic analysis (Fig. 2) as well as additional locations represented by the haplotypes

Species name assigned in this study^a^	GenBank Accession number	Country	State / Region	Location	Host	Date of collection	Collector / Supplier
*Lecanosticta acicola*	KJ938442	Japan	Shimane	Matsue, Hamanogi	*Pinus thunbergii*	Feb 2010	Suto Y
*L. acicola*	KJ938439	Mexico	Nuevo León	Iturbide, Bosque Escuela	*Pinus halepensis*	May 2010	Marmolejo JG
*L. acicola*	KJ938440	Mexico	Nuevo León	Iturbide, Bosque Escuela	*Pinus halepensis*	May 2010	Marmolejo JG
*L. acicola*	KJ938441	Mexico	Nuevo León	Iturbide, Bosque Escuela	*Pinus halepensis*	May 2010	Marmolejo JG
*L. acicola*	KJ938438	USA	Maine	York, Lyman	*Pinus strobus*	Jun 2011	Ostrofsky W
*L. acicola*	KJ938443	USA	Mississippi	Harrison County	*Pinus palustris*	Oct 2012	Bartlett B, Burdine C
*L. acicola*	KJ938444	USA	Mississippi	Harrison County	*Pinus palustris*	Oct 2012	Bartlett B, Burdine C
*L. acicola*	KJ938450	USA	Mississippi	Harrison County	*Pinus palustris*	Oct 2012	Bartlett B, Burdine C, Roberds J
*L. acicola*	KJ938451	USA	Mississippi	Harrison County	*Pinus palustris*	Oct 2012	Bartlett B, Burdine C
*Lecanosticta variabilis*	KJ938445	Guatemala	Alta Verapaz	Santa Cruz Verapaz, near Tactíc	*Pinus oocarpa*	Oct 2010	Barnes I
*L. variabilis*	KJ938446	Guatemala	Alta Verapaz	Santa Cruz Verapaz, near Tactíc	*Pinus oocarpa*	Oct 2012	Barnes I
*L. variabilis*	KJ938447	Mexico	Nuevo León	Piñal de los Amoles, Querétaro	*Pinus* sp.	2011	Kunte L
*L. variabilis*	KJ938448	Mexico	Nuevo León	Iturbide, Bosque Escuela	*Pinus halepensis*	May 2010	Marmolejo JG
*L. variabilis*	KJ938449	Mexico	Nuevo León	Galeana, Cerro del Potosí	*Pinus arizonica* var. *stormiae*	Apr 2010	Marmolejo JG
Countries, regions, locations and hosts represented by the above isolates^b^							
	the same as KJ938438	Austria	Lower Austria	Hollenstein an der Ybbs	*Pinus mugo*	Oct 2004	Kirisits T, Barnes I
	the same as KJ938438	Austria	Lower Austria	Opponitz	*Pinus mugo*	2010	Hintsteiner M
	the same as KJ938438	Austria	Lower Austria	Saimannslehen	*Pinus* sp.	2010	Hintsteiner M
	the same as KJ938438	Austria	Lower Austria	Sankt Gallen	*Pinus mugo*	2010	Hintsteiner M
	the same as KJ938438	Austria	Lower Austria	Steyer, Pestalozzistraße	*Pinus mugo*	2010	Hintsteiner M
	the same as KJ938438	Austria	Lower Austria	Waidehofen an der Ybbs	*Pinus mugo*	Aug 2010	Janoušek J
	the same as KJ938438	Austria	Upper Austria	Gmunden	*Pinus nigra*	Jun 2012	Kirisits T
	the same as KJ938438	Canada	Québec	Demers-Centre	*Pinus strobus*	Jun 2011	Harvey L
	the same as KJ938438	Canada	Québec	Lake Aberdeen	*Pinus strobus*	Jun 2011	Harvey L
	the same as KJ938438	Canada	Québec	Lake Pinseault	*Pinus strobus*	Jun 2011	Harvey L
	the same as KJ938438	Canada	Québec	Montréal	*Pinus mugo*	Jun 2011	Harvey R
	the same as KJ938438	Canada	Québec	Waltham	*Pinus strobus*	Jun 2011	Harvey L
	the same as KJ938442	China	Fujie		*Pinus elliottii*	1988	Zheng-Yu H
	the same as KJ938451	Colombia	Refocosta L-75	Villanueva, Casanare	*Pinus caribaea*	Mar 2011	Rodas C, Barnes I
	the same as KJ938438	Croatia		Zadar	*Pinus halapensis*	Sep 2009	Diminic D
	the same as KJ938438	Czech Republic	Southern Bohemia	Borkovická Blata	*Pinus uncinata* subsp. *uliginosa*	Oct 2011	Janoušek J
	the same as KJ938438	Czech Republic	Southern Bohemia	Červená Blata	*Pinus uncinata* subsp. *uliginosa*	Aug 2009	Dvořák M, Janoušek J
	the same as KJ938438	Estonia	Harju maakond	Tallin	*Pinus ponderosa*	Jul 2008	Cech T
	the same as KJ938451	France	Pyrénées-Atlantiques		*Pinus radiata*	2012	Kersaudy E, Ioos R
	the same as KJ938438	Germany	Bavaria	Grassau	*Pinus mugo*	2000	Blaschke FR, Wulf
	the same as KJ938438	Germany	Bavaria	Murnau	*Pinus mugo*	Feb 2010	Nannig A
	the same as KJ938438	Germany	Bavaria	Murnauer Filze	*Pinus mugo*	Nov 2011	Nannig A
	the same as KJ938438	Germany	Bavaria	Pfrűhlmoos	*Pinus mugo*	Nov 2011	Nannig A
	the same as KJ938438	Italy	Brecia	Gardone	*Pinus mugo*	Jun 2008	Cech T
	the same as KJ938438	Lithuania	Klaipėdský kraj	Curonian Spit, Juodkrante	*Pinus mugo*	2010	Markovskaja S
	the same as KJ938438	Slovenia	Upper Carniola	Bled	*Pinus mugo*	Jul 2009	Jurc D
	the same as KJ938442	South Korea	Naju	Sanpo-myeon	*Pinus thunbergii*	2010	KACC, Seo ST
	the same as KJ938451	Spain	Cantabria	San Sebastián de Garabandal	*Pinus radiata*	Oct 2012	Jankovský L, Janoušek J
	the same as KJ938438	Switzerland	Canton St Gallen	Walensee	*Pinus mugo*	Oct 1999	Wulf
	the same as KJ938438	USA	Maine	Androscoggin, Leeds	*Pinus strobus*	Jun 2011	Ostrofsky W
	the same as KJ938438	USA	Maine	Piscataquis, Sangerville	*Pinus strobus*	Jun 2011	Weimer J
	the same as KJ938438	USA	Maine	York, Lyman	*Pinus strobus*	Jun 2011	Ostrofsky W
	the same as KJ938438	USA	Michigan	Wexford County, Springville Township	*Pinus sylvestris*	2011	Odonnell J
	the same as KJ938444	USA	Mississippi	Harrison County	*Pinus palustris*	Oct 2012	Bartlett B, Burdine C, Roberds J
	the same as KJ938438	USA	New Hampshire	Hillsboro, Fox State Park	*Pinus strobus*	Jun 2011	Weimer J
	the same as KJ938438	USA	New Hampshire	Merrimack, Black Water Reserve	*Pinus strobus*	Jun 2011	Weimer J
	the same as KJ938438	USA	New Hampshire	Merrimack, Hopkinton-Everett	*Pinus strobus*	Jun 2011	Weimer J
	the same as KJ938438	USA	Vermont	Washington, Waterbury	*Pinus strobus*	Jun 2011	Lackey J
	the same as KJ938438	USA	Vermont	Windsor, Bethel	*Pinus strobus*	Jul 2011	Munck I
	the same as KJ938438	USA	Wisconsin	Merrillan	*Pinus sylvestris*	Apr 2010	Stanosz G

^a^*Lecanosticta variabilis* was previously identified as *L. acicola* but is now defined as a new species

^b^Information adapted from Janoušek et al. (2016), Table S1

Three separate analyses were performed for each of the six datasets: Maximum Parsimony (MP), Maximum Likelihood (ML) and Bayesian inference (BI). The MP analysis were performed with the software package PAUP* 4.0b10 (Swofford 2003). Gaps were treated as a fifth character state. One thousand random stepwise addition heuristic searches were performed with tree-bisection-reconnection (TBR) as the branch-swapping algorithm. Uninformative characters were excluded and the consistency index (CI), homoplasy index (HI), rescaled consistency index (RC), retention index (RI) and tree length (TL) were determined for the resulting trees (Table 4). The confidence levels were estimated by performing 1000 bootstrap replicates.

**Table 4:** PCR amplification size, phylogenetic data and the substitution models used in the phylogenetic analysis for each gene region and for the combined datasets

	ITS	*TEF*1	*BT*1	*MS204*	*RPB*2	Combined datasets
Approximate amplicon size (bp)	550	520	420	760	940	–
Number of taxa analysed	153	147	111	91	105	76
Aligned characters (bp)	734	586	440	785	929	3344
Number of parsimony-uninformative characters	621	143	357	519	538	2438
Number of parsimony-informative characters	114	423	82	266	371	1121
Number of trees retained	108	396	1	2448	420	100
Consistency index	0.865	0.499	0.739	0.791	0.738	0.607
Homoplasy index	0.135	0.501	0.261	0.209	0.262	0.393
Rescaled consistency index	0.850	0.459	0.703	0.748	0.696	0.555
Retention index	0.982	0.919	0.951	0.946	0.943	0.914
Tree Length	163	1675	138	546	722	2642
Substitution model	TPM2uf + G	GTR + G	GTR + G	TVM + G	TrN + G	GTR + G

In order to determine the ML and BI, the best fit substitution model for each of the data sets were determined using jModelTest 0.1.1 (Posada 2008). Maximum likelihood analysis was performed with the program PhyML 3.0 (Guindon et al. 2010). The confidence levels were estimated with 1000 bootstrap replicates.

MrBayes 3.1.2 (Ronquist et al. 2012) was used to determine the BI for each data set by applying the Markov Chain Monte Carlo (MCMC) method. For each dataset, four independent MCMC chains were randomly started and run for six million generations, applying the best substitution model determined by jModelTest 0.1.1. Trees were sampled every 100 generations. Burn-in values were determined using Tracer 1.6 (Rambaut et al. 2014) by comparing the log likelihoods. Trees sampled in the burn-in phase were discarded. The remaining trees were used to construct majority rule consensus trees and to determine posterior probabilities for the tree topology.

### Morphological characterization

Cultures were grown on 2% Malt Extract Agar (MEA), Oatmeal Agar (OA) and Potato Dextrose Agar (PDA) (Crous et al. 2009b; Quaedvlieg et al. 2012) at 20 °C for 2 wk. in darkness in order to examine the morphology and colour of the cultures of each species. Cultures on MEA were used for microscopic measurements of the conidiophores, conidiogenous cells and conidia. Slides were mounted in SABAX water (Adcock Ingram, Midrand, South Africa) for microscopy and examined using a Zeiss Axioskop 2 Plus compound microscope (Zeiss, Oberkochen, Germany). Photographic images were captured with a Nikon DS-Ri2 camera with the NIS Element BR v4.3 software package (Nikon, Tokyo, Japan). Up to 50 measurements of each morphologically characteristic structure was taken for each ex-type isolate and ten measurements were made for each of the paratypes examined. The mean, standard deviation, minimum and maximum were calculated for each morphological structure and the measurements presented as (minimum–) (mean – standard deviation) – (mean + standard deviation) (−maximum) for the conidia and conidiogenous cells. For the conidiophores, the maximum observed length was indicated together with the width as (minimum–) (mean) (−maximum).

**Table 5:** Specimens for which the morphology was examined for the description of *Lecanosticta jani, L. pharomachri, L. tecunumanii* and *L. variabilis*

Species	CMW number^a^	Status of specimen	Herbarium specimen^b^	Ex-type isolates^c^
*Lecanosticta jani*	CMW 38950^d^	Paratype	PREM 62186	CBS 144446
	CMW 38958^d^	Holotype	PREM 62185	CBS 144456
	CMW 48831^e^	Paratype	PREM 62187	CBS 144447
	CMW 51058^d^	Additional material examined		
	CMW 51059^d^	Additional material examined		
	CMW 51143^e^	Additional material examined		
	CMW47109^e^	Additional material examined		
*Lecanosticta pharomachri*	CMW 37136	Holotype	PREM 62188	CBS 144448
	CMW 38947	Paratype	PREM 62189	CBS 144695
	CMW 38974	Paratype	PREM 62190	CBS 144449
	CMW 38976	Additional material examined		
	CMW 51053	Additional material examined		
	CMW 51054	Additional material examined		
*Lecanosticta tecunumanii*	CMW 46805	Holotype	PREM 62191	CBS 144450
	CMW 46812	Paratype	PREM 62193	CBS 144452
	CMW 49403	Paratype	PREM 62192	CBS 144451
*Lecanosticta variabilis*	CMW 42205	Holotype	PREM 62196	CBS 144453, IMI 281561
	CMW 37125	Paratype	PREM 62194	CBS 144454
	CMW 36809	Paratype	PREM 62195	CBS 144455
	CMW 45425	Additional material examined	CBS H-21112	CBS 133789
	CMW 37129	Additional material examined		

^a^*CMW* Culture collection of the Forestry and Agricultural Biotechnology Institute (FABI), University of Pretoria, South Africa; ^b^The herbarium deposits are dried cultures that serve as holotype and paratype specimens. PREM = The dried herbarium collection of the South African National Collection of Fungi, Mycology Unit, Biosystematics Division, Plant protection Institute, Agricultural Research Council, Pretoria, South Africa; ^c^The ex-type cultures are living cultures linked to the holotype and paratype specimens. CBS = The culture collection of the Westerdijk Fungal Biodiversity Institute, Utrecht, The Netherlands; IMI = The UK National Fungus Collection maintained by CABI Bioscience, Egham, UK; ^d^ *Lecanosticta jani* cultures with the Type 2 morphology; ^e^ *Lecanosticta jani* cultures with the Type 1 morphology

Temperature requirements for growth in culture was studied on representative isolates selected for each of the novel species. Four by four millimeter blocks of each culture were plated, in triplicate, onto the centres of 2% MEA plates per temperature (10, 15, 20, 25, and 30 °C) and incubated in darkness. The diameters of each colony were recorded weekly along perpendicular axes for 4 wk. The colour and shape of each colony was recorded after 2 wk. of growth at 20 °C. Culture colour was determined using Rayner’s colour chart (Rayner 1970).

### Accession of cultures and types

Holotype specimens of the new species, which are dried cultures, are deposited in the National Mycological Herbarium in Pretoria (PREM). Cultures are deposited in the culture collection (CBS) of the Westerdijk Fungal Biodiversity Institute, Utrecht, The Netherlands, and ex-type cultures, as well as all other isolates included in this study, are maintained in the culture collection (CMW) of the Forestry and Agricultural Biotechnology Institute (FABI) in Pretoria, South Africa (Table 5).

## RESULTS

### Fungal collections

Twenty-six isolates or DNA samples were obtained from culture collections to include in the study. An additional 127 isolates of putative *Lecanosticta* species were obtained from symptomatic needles collected from 36 different trees in Guatemala, Nicaragua and Honduras (Table 1). In Guatemala, 22 isolates were obtained from *Pinus oocarpa, P. maximinoi,* and *P. tecunumanii* needles that were collected in the Alta Verapaz District, 16 isolates were obtained from *P. oocarpa* needles collected in Chiquimula, 35 isolates from *P. pseudostrobus* needles collected in the Chimaltenango District in the Tecpán Municipality, eight isolates from *P. tecunumanii* needles collected in the Baja Verapaz District, 29 isolates from *P. tecunumanii* and *P. oocarpa* needles collected in the Jalapa District, and seven isolates from *P. maximinoi* needles in Coban and other regions (Table 1). Two isolates were obtained from *P. oocarpa* needles collected in Honduras and eight isolates were made from *P. oocarpa* needles collected in Matagalpa, Nicaragua.

### DNA extraction and sequencing

The ITS and *TEF*1 regions were sequenced for all 153 isolates obtained and the *BT*1, *MS204* and *RPB*2 regions were sequenced for 127 representatives of all monophyletic groups identified in the generated ITS and *TEF*1 phylogenetic trees. The selected representatives included all of the closely related *Mycosphaerellaceae* isolates, all the isolates that did not group with known *Lecanosticta* species, and a selection of isolates that grouped with known *Lecanosticta* species (Table 1). PCR fragments of approximately 550 bp were generated for ITS, 520 bp for *TEF*1, 420 bp for *BT*1, 760 bp for *MS204* and 940 bp for *RPB*2. The amplification success of the *TEF*1, *BT*1, *MS204* and *RPB*2 gene regions varied for the isolates that were selected and the amplification success rate of *TEF*1 was 88.2%, *BT*1 was 87.4%, *MS204* was 71.7 and 82.7% for the *RPB*2 region (Table 2). The *BT*2 region did not amplify well across species of *Lecanosticta*. The amplification success rate and subsequent sequencing of the *BT*2 region using the T1 and β-Sandy-R primer pair, as well as Bt2a and Bt2b was very poor and further analysis of the *BT*2 region was abandoned.

### Phylogenetic analyses

**Fig. 1:**
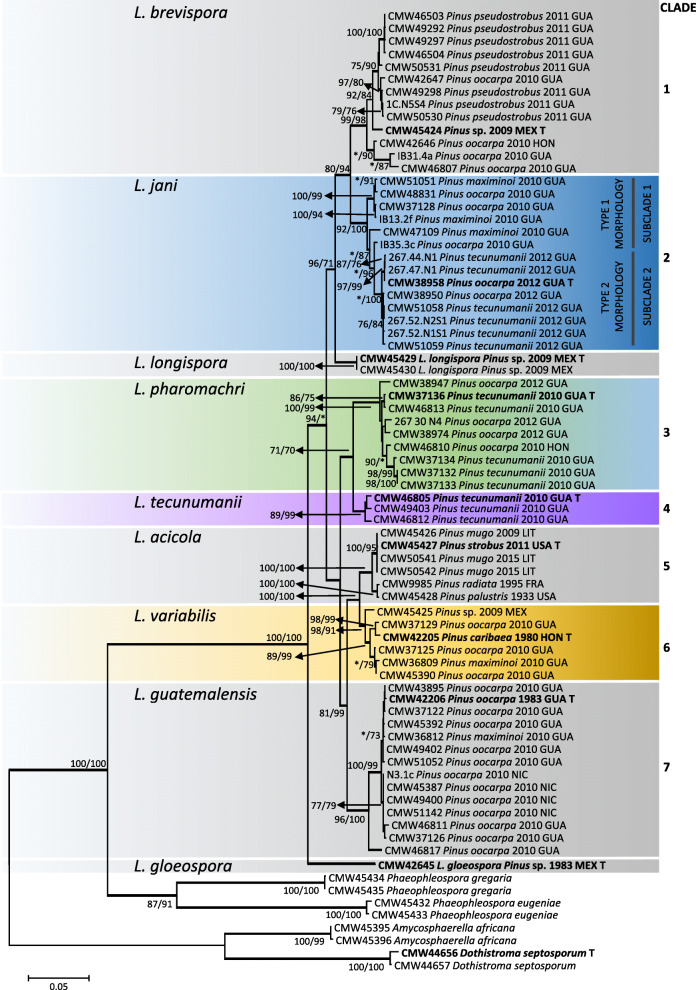
Maximum likelihood tree representing the five known and four novel species of *Lecanosticta* generated from the combined data of the ITS, *TEF*1, *BT*1, *MS204* and *RPB*2 gene regions. MP bootstrap support (> 70%) are indicated first, followed by ML bootstrap values (MP/ML, * = insignificant value). Bold branches indicate BI values > than 0.95. *Dothistroma septosporum* was used as the outgroup taxa. The indicated clades are referred to in the text. All represented type species are indicated in bold and with a “T”

**Fig. 2:**
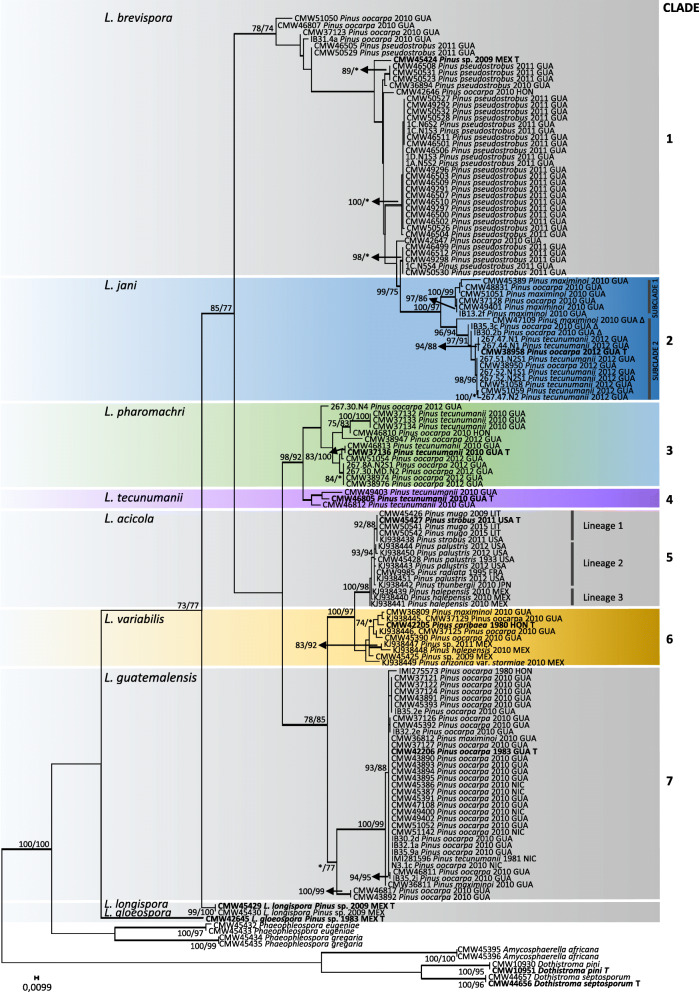
Maximum likelihood tree representing the five known and four novel species of *Lecanosticta* generated from the *TEF*1 region. MP bootstrap support (> 70%) are indicated first, followed by ML bootstrap values (MP/ML, * = insignificant value). Bold branches indicate BI values > than 0.95. *Dothistroma* species were used as the outgroup taxa. All represented type species are indicated in bold and with a “T”. Clades indicated on the left correspond with the clades in Fig. 1. Within the *L. jani* clade a “∆” next to the isolate indicates that the isolate either exhibits Type 2 morphology and groups with Subclade 1, or, exhibits Type 1 morphology and groups with Subclade 2

For the analyses, the datasets of the ITS region consisted of 153 taxa with 734 aligned nucleotides including gaps; the *TEF*1 dataset consisted of 147 taxa with 586 aligned nucleotides, the *BT*1 dataset consisted of 111 taxa with 440 aligned nucleotides; the *MS204* dataset consisted of 91 taxa with 785 aligned nucleotides, and the *RPB*2 dataset consisted of 105 taxa with 929 aligned nucleotides, all including gaps. The PHT test yielded a *P* value = 0.01 and therefore the five datasets were considered incongruent. However, it was previously argued that a P value > 0.01 did not reduce phylogenetic accuracy (Cunningham 1997) and a combined phylogenetic tree representing the five gene regions ITS, *TEF*1, *BT*1, *MS204* and *RPB*2 was constructed for presentation purposes (Fig. 1). The combined dataset consisted of 76 taxa with 3344 aligned nucleotides including gaps. Constant characters, parsimony-uninformative and informative characters, the consistency index (CI), homoplasy index (HI), rescaled consistency index (RC), retention index (RI) and tree length (TL) values for the maximum parsimony analyses are indicated in Table 4. For the parsimony analyses, 108 trees were retained for ITS, 396 for *TEF*1, 1 for *BT*1, 2448 for *MS204* and 420 for *RPB*2. The best fit substitution models for ML and BI were selected by Akaike Information Criterion (AIC) and are indicated in Table 4. A 10% burn-in value was selected in the BI analysis for each of the data matrices for each of the analyses. Because the MP, ML and BI analysis all resulted in similar tree topologies, the ML trees were selected and chosen for presentation (Figs. 1 and 2, Additional file 1: Figure S1, Additional file 2: Figure S2, Additional file 3: Figure S3 and Additional file 4: Figure S4).

Phylogenetic analyses of the combined dataset (Fig. 1), ITS (Additional file 1: Figure S1), *TEF*1 (Fig. 2) and *MS204* (Additional file 3: Figure S3) consistently grouped the isolates sequenced in this study into seven distinct clades. The clades in Fig. 2 and Additional file 1: Figure S1, Additional file 2: Figure S2, Additional file 3: Figure S3 and Additional file 4: Figure S4 are labelled according to the clades assigned in Fig. 1. In the case of *RPB*2 (Additional file 4: Figure S4) Clades 1–4, and 7 were also present but Clades 5 and 6 were not distinct from each other for this particular gene region. In the case of *BT*1 (Additional file 2: Figure S2), Clades 3, 5 and 6 could not be distinguished from each other. None of the isolates grouped with the types of *L. gloeospora* or *L. longispora*.

Forty-two of the isolates from Central America grouped in Clade 1 based on the ITS analysis (Additional file 1: Figure S1) and were identified as *Lecanosticta brevispora*. This was the most common species identified from the Central American collection and most isolates were from Chimaltenango on *Pinus pseudostrobus*. The pathogen was also isolated from *P. oocarpa* needles near Jalapa as well as near Tactíc in Guatemala and in Honduras*.* This clade was well supported for all five of the gene regions analysed.

Twenty-seven isolates grouped into Clade 2 in the ITS analyses (Additional file 1: Figure S1) and represent an undescribed species. Clade 2 resolved into two subclades in the five gene analyses. Subclade 1 was mostly isolated from Chiquimula and Alta Verapaz in Guatemala on *P. oocarpa, P. maximinoi* and *P. tecunumanii* as well as from *P. oocarpa* in Nicaragua. Isolates collected in Jalapa in Guatemala mostly grouped into Subclade 2. However, the topology of isolates CMW 47109 (Subclade 1 on Additional file 1: Figure S1, Additional file 3: Figure S3, Additional file 4: Figure S4; Subclade 2 on Fig. 2), CMW 51059 (Subclade 1 on Additional file 1: Figure S1, Additional file 3: Figure S3, Additional file 4: Figure S4), IB30.2b (Subclade 1 on Additional file 1: Figure S1, Additional file 3: Figure S3; Subclade 2 on Fig. 2) and IB30.2b (Subclade 1 on Additional file 1: Figure S1, Additional file 3: Figure S3, Additional file 4: Figure S4; Subclade 2 on Fig. 2) changed in the two subclades depending on the gene region analysed (Fig. 2, Additional file 1: Figure S1, Additional file 3: Figure S3, Additional file 4: Figure S4). Furthermore, the two subclades were not well supported for the *BT*1 gene region. Therefore, the two subclades are treated here as representing a single species.

Clade 3 also represented an undescribed *Lecanosticta* species. This clade included 11 isolates from *P. oocarpa* in Jalapa, Guatemala, one isolate from *P. oocarpa* in Honduras, as well as five isolates collected from Baja Verapaz in Guatemala on *P. tecunumanii.* This clade had high bootstrap support for *TEF*1, *MS204* and *RPB*2 but was not well supported in the ITS and *BT*1 gene regions. Three isolates collected from different needles on a single *P. tecunumanii* tree in Baja Verapaz in Guatemala grouped together in Clade 4 and represent another undescribed species. With the exception of *BT*1, Clade 4 was statistically well supported in all the gene regions that were analysed.

Clade 5 accommodated sequences representing nine of the 14 known *TEF*1 haplotypes of *L. acicola* identified by Janoušek et al. (2016). These *TEF*1 haplotypes represent isolates collected from North America (Canada, USA, and Mexico), South America (Colombia), Europe (Spain, France, Switzerland, Slovenia, Lithuania, Italy, Germany, Estonia, Czech Republic, Croatia, and Austria) and Asia (South Korea, Japan, and China) (Table 3). This clade was clearly distinct from other clades in the ITS, *TEF*1, *BT*1 and *MS204* phylogenetic analysis and statistically well supported in the ITS, *TEF*1, and *MS204* analyses. Clade 5 included the ex-type of *L. acicola* and therefore is that species. None of the isolates from Central America obtained in the present study grouped with this clade in any of the gene regions analysed.

The remaining five assigned *L. acicola TEF*1 haplotypes considered by Janoušek et al. (2016), grouped together in Clade 6. This was together with an isolate obtained from *P. caribaea* in Honduras collected in 1983 (Evans 1984), four isolates obtained in the present study from Guatemala on *P. oocarpa* and *P. maximinoi,* and an isolate previously identified as *L. acicola* from Mexico on an unknown *Pinus* species (Quaedvlieg et al. 2012). In the present study, Clade 6 is treated as a novel taxon. The ITS, *TEF*1, *BT*1 and *MS204* gene regions clearly distinguish Clades 5 and 6, however, *RPB*2 was not effective in resolving these two groups.

The second most abundant species collected in this study was *Lecanosticta guatemalensis*, represented by Clade 7 in the phylogenetic analyses. This clade was well supported in all five gene regions that were analysed. A total of 37 isolates from our collection grouped together with *L. guatemalensis* based in the ITS and *TEF*1 analyses. *Lecanosticta guatemalensis* was identified on *P. maximinoi* and *P. oocarpa* in various regions of Guatemala, as well as on *P. oocarpa* in Nicaragua. Isolates that had previously been collected in Nicaragua and Honduras and that were identified as *L. acicola* by Evans (1984) based on morphological characteristics also grouped with *L. guatemalensis* in the present study*.*

### TAXONOMY

Using phylogenetic analyses, 51 of the *Lecanosticta* isolates obtained from Guatemala, Honduras and Nicaragua, one isolate obtained from CBS, and one isolate obtained from IMI, were found to include four undescribed species. These are described below as follows:

**Lecanosticta jani** van der Nest, M.J. Wingf. & I. Barnes, **sp. nov.**

**Fig. 3:**
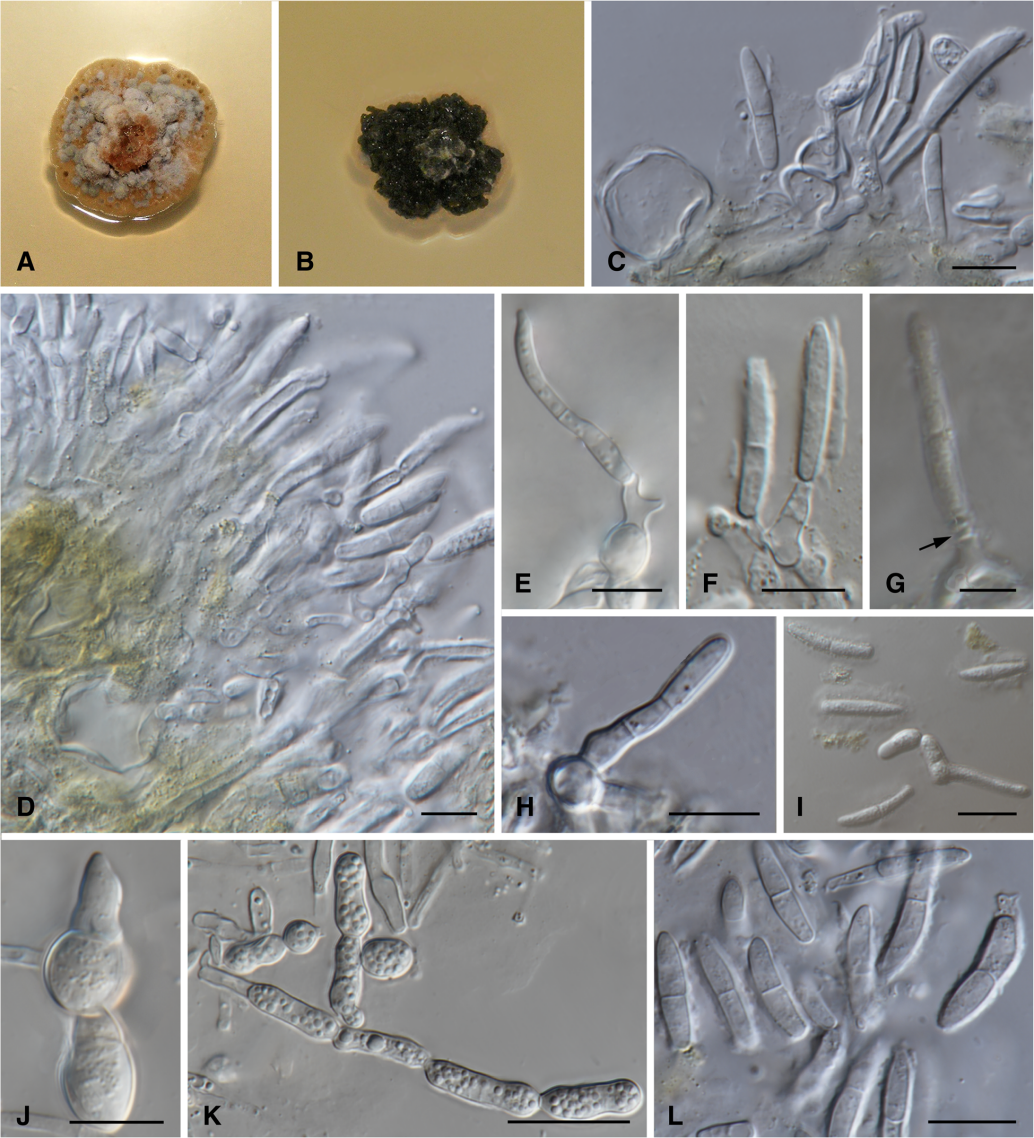
*Lecanosticta jani* (CMW38958; CMW38950; CMW48831; CMW47109; CMW51058; CMW51143) **a**-**b** Two wk. old colonies on MEA. A represents Type 1 colonies (CMW38950) and B represents Type 2 colonies (CMW48831). **c-h** Conidiogenous cells giving rise to conidia on MEA, with swollen globose basal cells of the conidiophores in E, F and H as well as annelations (see arrow) in G. **i-k** Swollen conidiogenous cells and conidia on MEA. Note endospore formation and germination in I. **l** Conidia on MEA. Bars: K = 50 μm; C-F and H-L = 10 μm; G = 5 μm

*Notes*: *Lecanosticta jani* resolved in a distinct clade (Clade 2, Figs. 1 and 2, Additional file 1: Figure S1, Additional file 2: Figure S2, Additional file 3: Figure S3 and Additional file 4: Figure S4) based on all five gene regions considered. This clade divides into two subclades that were mostly represented by isolates obtained from Alta Verapaz and Chiquimula in Guatemala as well as in Nicaragua in subclade 1 and isolates obtained from Jalapa in Guatemala in subclade 2. Jalapa isolates all had the Type 2 morphology and the dark colour was associated with conidial production. Type 1 isolates produced few spores after 2 wk. The optimal growth temperature and growth rates were different for the two isolate types. However, the topology of some isolates changed between the two subclades depending on the gene region that is analysed and therefore the subclades are treated as one species. The morphological variation suggests that the two types could represent two ecotypes.

MycoBank MB 826875. (Fig. 3)

*Etymology*: The name is derived from Janus, the Roman god of gates and doorways having two faces or sides, and refers to the variable culture morphology ranging from light pink and fluffy to dark olive green and mucoid.

*Diagnosis*: *Lecanosticta jani* can be distinguished from the closely related *L. brevispora* by the distinct globose basal cells on the conidiophores that are mostly observed on MEA.

*Type*: **Guatemala**: Jalapa, Finca la Soledad, Mataquescuintla, on needles of *Pinus oocarpa*, 20 Sept 2012, *I. Barnes* (PREM 62185 – holotype; CMW 38958 = CBS 144456 – ex-type culture).

*Description*: *Sexual morph* unknown. *Conidiomata* isabelline to vinaceous brown on MEA. *Conidiophores* subcylindrical, often with a swollen globose basal cell, densely aggregated, honey to hyaline, smooth to verruculose, unbranched or branched at base, often encased in a yellow to light brown mucoid sheath, to 82 μm in length, 4.5–7.0 μm diam. *Conidiogenous cells* terminal, integrated, subcylindrical, honey to hyaline, smooth to verruculose, proliferating several times percurrently with visible annelations near apex, septate or aseptate, (8.5–)16.5(− 24.0) × (3.0–)4.5(− 6.5) μm. *Conidia* solitary, sub-cylindrical to narrowly fusoid-ellipsoidal, with subobtusely rounded apex, base truncate, brown, verruculose, frequently with mucoid sheath, two distinct sizes with conidial type one more abundant than conidial type two. *Conidial type 1*: 1–2-septate, base (1.5–)2.0–2.5(− 3.5) μm diam, (9.5–)14.5–21.5(− 30.0) × (2.0–)2.5–3.5(− 4.0) μm. *Conidial type 2*: 1–3-septate, base (1.5–)2.0–2.5(− 3.0) μm diam, (26.5–)30.5–37.0(− 38.0) × (2.0–)2.5–3.0(− 3.5) μm.

*Culture characteristics*: Colonies with two distinct morphologies. One type (Type 1), flat to somewhat erumpent, spreading with flat to fluffy aerial mycelium. A second type (Type 2) erumpent, mucoid and shiny, with irregular form and undulate to filiform edges. On MEA, the surface of Type 1 isolates pale to rosy vinaceous, reverse flesh to peach coloured. Type 2 isolates citrine to isabelline, reverse olivaceous to fuscuous black (Fig. 3). On PDA, Type 1 surface rosy vinaceous to peach in centre with dark brown edge, isabelline in reverse. Type 2, surface dark olivaceous with fuscious black centres and tufts of isabelline mycelium at edges, dark isabelline in reverse. On OA, Type 1 surface dirty white to pale vinaceous, fluffy mycelia to flat growth. Type 2 surface flat with smooth edge, fuscious black in centre at the point of inoculation with light apricot surrounding mycelium. *Growth characteristics:* optimal growth temperature for Type 1 isolates 25 °C, after 4 wk., colonies at 10, 15, 20, 25 and 30 °C reached maximum of 10.5, 22, 32, 32 and 10 mm respectively, with mean growth rate of 2.1, 5.1, 6.9, 7 and 1.8 mm / wk. respectively. Type 2 isolates optimal growth temperature 20 °C, after 4 wk., colonies at 10, 15, 20, 25 and 30 °C reached maximum of 12.5, 17, 29.5, 22 and 4.5 mm, with mean growth of 2.1, 3.3, 5.5, 5 and 1 mm / wk. respectively.

*Additional material examined*: **Guatemala:** Alta Verapaz, Santa Cruz Verapaz, near Tactíc, on needles of *Pinus oocarpa*, 21 Oct 2010, *I. Barnes* (culture CMW47109); *loc. cit. I. Barnes* (PREM 62187; CMW 48831 = CBS 144447 – culture); Jalapa, Finca la Soledad, Mataquescuintla, on needles of *Pinus oocarpa*, 20 Sept 2012, *I. Barnes* (PREM 62186, CMW 38950 = CBS 144446 – culture); Jalapa, Finca la Soledad, Mataquescuintla, on needles of *Pinus tecunumanii*, 20 Sept 2012, *I. Barnes* (cultures CMW 51058, CMW 51059). -**Nicaragua:** Matagalpa, on needles of *Pinus oocarpa*, 20 June 2011, *I. Barnes* (culture CMW 51143).

**Lecanosticta pharomachri** van der Nest, M.J. Wingf. & I. Barnes, **sp. nov.**

**Fig. 4:**
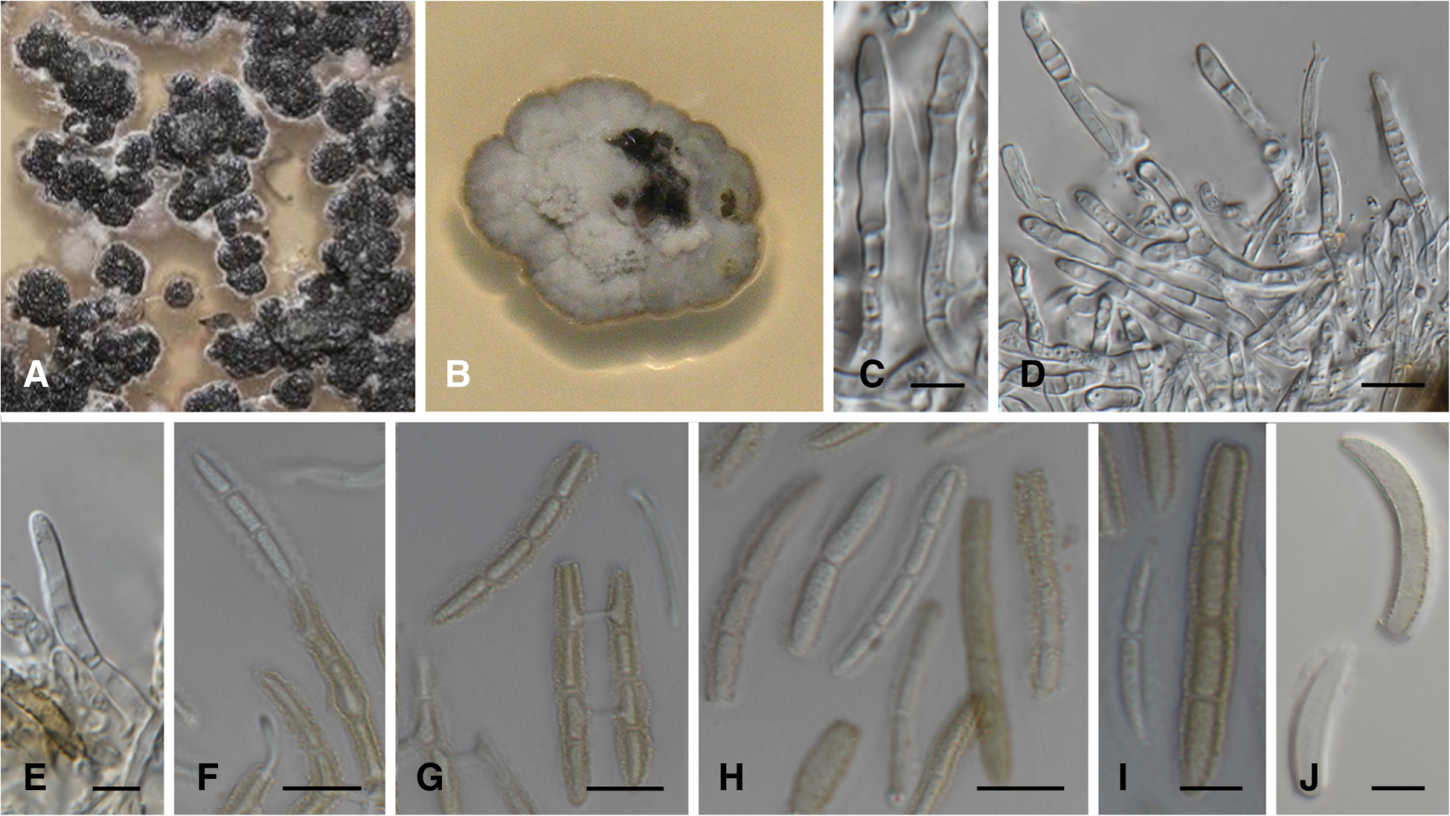
*Lecanosticta pharomachri* (CMW 37136; CMW38947)*.* **a**, **b** Two wk. old colonies on MEA. **c**-**e** Conidiogenous cells giving rise to conidia on MEA. **f**, **g** Conjugation tube formation between conidia as well as conidia bearing smaller conidial cells. **h-j** Variation in conidia on MEA. Bars: D, F-H and J = 10 μm; C, E and I = 5 μm

MycoBank MB 826876. (Fig. 4)

*Etymology*: The epithet refers to the Resplendid Quetzal (*Pharomachrus mocinno*), which is the national bird of Guatemala and the spirit bird/companion of Tecún Umán; a Guatemalan legend.

*Diagnosis*: *Lecanosticta pharomachri* is distinguished from the other taxa in the genus by all five gene regions investigated but especially by sequences of *TEF*1, *MS204* or *RPB*2. Conidia are also larger than those of *L. guatemalensis* and similar to *L. acicola* but differ from these species in that the conidia are frequently surrounded by a thick mucoid sheath and are mostly straight.

*Type*: **Guatemala:** Baja Verapaz, San Jerónimo, Salamá, on needles of *Pinus tecunumanii*, Nov 2010, *I. Barnes* (PREM 62188 – holotype; CMW 37136 = CBS 144448 – ex-type cultures).

*Description*: *Sexual morph* not observed. *Conidiomata* dark vinaceous brown on MEA. *Conidiophores* subcylindrical to cylindrical, densely aggregated, vinaceous brown to hyaline, smooth to verruculose, unbranched or branched at base, often encased in a light brown mucoid sheath, to 45 μm in length, 2.5–4.0 μm diam. *Conidiogenous cells* terminal, integrated, subcylindrical to cylindrical, luteus brown to hyaline, smooth to verruculose, surrounded by mucilage, holoblastic, proliferating several times percurrently with visible annelations near apex, septate or aseptate, (6.5–)9.5–13.5(− 16.0) × (1.5–)2.0–2.5(− 3.0) μm. *Conidia* released in a greenish olivaceous to honey mass, solitary, straight to slightly curved, cylindrical, with subobtusely rounded apex, base truncate, guttulate, hyaline to light brown, verruculose, frequently with thick mucoid sheath, 1–3-septate, base (1.5–)2.0–3.0(− 3.5) μm diam, (21.0)25.0–34.0(− 49.0) × (2.5–)3.0–4.0(− 5.0) μm. Germ tubes observed between conidia as well as conidial budding - secondary conidia sometimes produced from apical cell, 0–2-septate.

*Culture characteristics*: Colonies flat to erumpent, form irregular with undulate edge, spreading with fluffy aerial mycelium at centers. On MEA, surface apricot to cinnamon with isabelline and rosy buff mycelial mat at centers, reverse isabelline to dark brick in centre with cinnamon to apricot edges. On PDA, surfaces rosy to pale vinaceous with light isabelline to greenish white edges, reverse isabelline with cream edges. On OA, surface dirty white to isabelline to dark brown, fluffy mycelium to flat growth. *Growth characteristics:* optimal growth temperature 20 °C, after 4 wk., colonies at 10, 15, 20, 25, and 30 °C reaching a maximum of 9, 17, 18.5, 18.5 and 8.5 mm diam, with mean growth rates of 1.9, 3.6, 4.6, 4.4, and 1.9 mm / wk. respectively.

*Notes*: Some of the isolates, including the ex-type strain, produced a luteus exudate that diffused into MEA after 4–6 wk. Conjugation tubes were reported previously in *L. acicola* cultures as well as in needles (Siggers 1950; Crosby 1966). Conjugation tubes were also observed in this species (Fig. 4g) in the present study. Endospores as described by Crosby (1966) were also observed in some conidia.

*Additional material examined*: **Guatemala**: Jalapa, Finca la Soledad, Mataquescuintla, on needles of *Pinus oocarpa*, 20 Sept 2012, *I. Barnes* (cultures CMW 38976, CMW 51053 and CMW 51054); *loc. cit*., *I. Barnes* (PREM 62189; CMW 38947 = CBS 144695 – culture; PREM 62190, CMW 38974 = CBS 144449 – culture).

**Lecanosticta tecunumanii** van der Nest, M.J. Wingf. & I. Barnes, **sp. nov.**

**Fig. 5:**
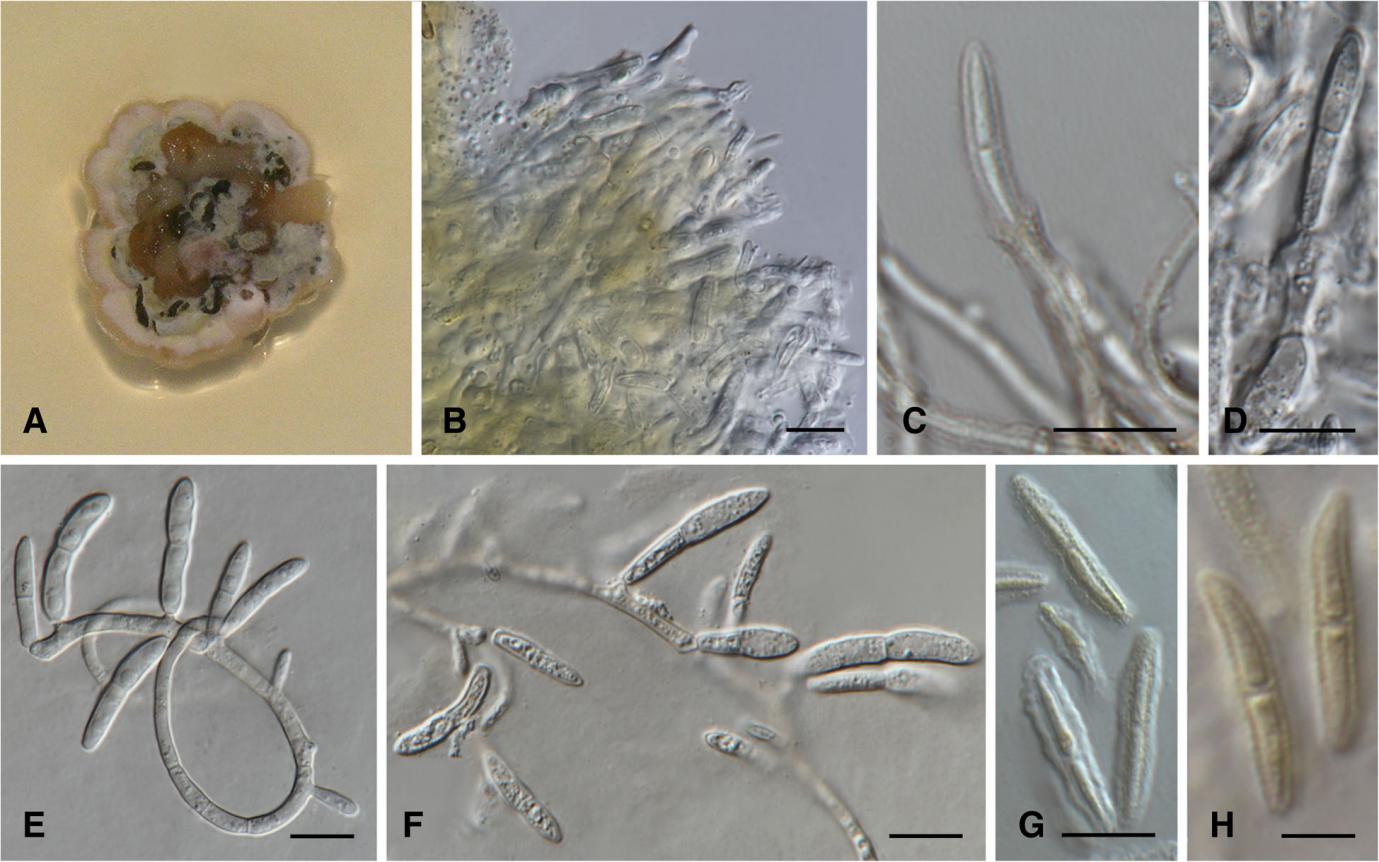
*Lecanosticta tecunumanii* (CMW46805; CMW46812). **a** Two wk. old colony on MEA**. b-d** Conidiogenous cells giving rise to conidia on MEA. **e-f** Micronematous conidiogenesis observed on MEA with conidia. **g-h** Uniseptate conidia with or without a mucoid sheath observed on MEA. Bars: B-G = 10 μm; H = 5 μm

MycoBank MB 826877. (Fig. 5)

*Etymology*: Name refers to the Guatemalan legend, Tecún Umán, and *Pinus tecunumanii*, the host plant from which the holotype was collected.

*Diagnosis*: *Lecanosticta tecunumanii* is distinguished from the other taxa by the ITS, *TEF*1, *MS204* and *RPB*2 gene regions. Morphologically, it is distinct in having only 1-septate conidia after 2 wk. of incubation on MEA, but 2-septate and 3-septate conidia are occasionally observed in older cultures.

*Type*: **Guatemala**: Baja Verapaz, San Jerónimo, Salamá, on needles of *Pinus tecunumanii*, Oct 2011, *I. Barnes* (PREM 62191 – holotype; CMW 46805 = CBS 144450 – ex-type cultures).

*Description*: *Sexual morph* not observed. *Conidiomata* isabelline to visaceous brown on MEA. *Conidiophores* cylindrical, densely aggregated, hyaline to pale yellow-brown, smooth to slightly verruculose, unbranched or branched at base, to 120 μm in length, 2.0–5.0 μm diam. *Conidiogenous cells* terminal or indeterminate, integrated or discrete, cylindrical, hyaline to honey, smooth to verruculose, proliferating several times percurrently with visible annelations near apex or micronematous, septate or aseptate, (5.0–)7.0–14.5(− 15.5) × (1.5–)2.0–2.5(− 3.0) μm. Micronematous cells (6–)10.5–18.5(− 27.0) × (2.0–)2.0–2.5(− 3.0) μm. *Conidia* solitary, straight to slightly curved, subcylindrical to fusiform, with subobtusely rounded or sharply pointed apex, base truncate, guttulate, smooth to granulate, hyaline to cream buff to light brown, occasionally enclosed in mucoid sheath, 1-septate, base (1.5–)1.5–2.0(− 2.0) μm diam., (14.5–)16.0–21.0(− 24.0) × (2.0–)2.5–3.0(− 3.5) μm.

*Culture characteristics*: Colonies somewhat erumpent, spreading with flat to fluffy aerial mycelium. On MEA, surface olivaceous to isabelline with rosy buff mycelial tufts, reverse isabelline. On PDA, surface rosy vinaceous to peach in centre with a dark brown edge, isabelline in reverse. On OA, surface dirty white to pale vinaceous, fluffy mycelia to flat peach growth. *Growth characteristics*: optimal growth temperature 25 °C, after 4 wk., colonies at 10, 15, 20, 25, and 30 °C reached maximum of 9, 15.5, 24, 24, and 4.5 mm, with mean growth of 2.2, 3.8, 5.3, 5.7, and 1.1 mm / wk. respectively.

*Notes*: Micronematous conidiogenesis (Fig. 5E - F), observed more frequently than distinct conidiophores in culture.

*Additional material examined*: **Guatemala**: Baja Verapaz, San Jerónimo, Salamá, on needles of *Pinus tecunumanii*, Oct 2011, *I. Barnes* (PREM 62192, CMW 49403 = CBS 144451 – culture; PREM 62193, CMW 46812 = CBS 144452 – culture).

**Lecanosticta variabilis** van der Nest, M.J. Wingf. & I. Barnes, **sp. nov.**

**Fig. 6:**
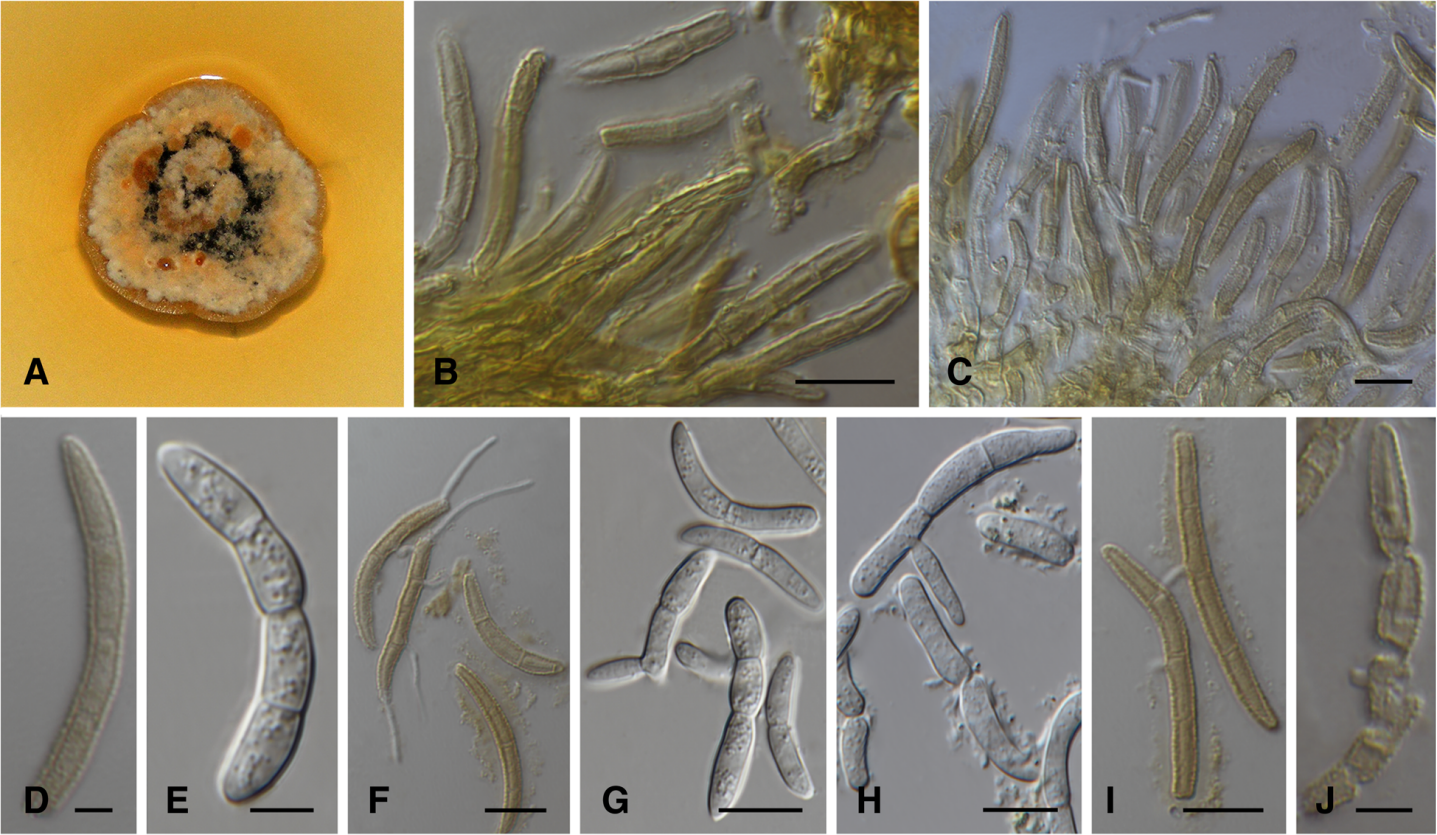
*Lecanosticta variabilis* (CMW42205; CMW37125). **a** Colony on MEA with luteus exudate diffusing into medium. **b-c** Conidiogenous cells giving rise to conidia on MEA. **d-h** Various conidial shapes and sizes on MEA. **f** Germinating conidia on MEA. **g-h** Swollen conidial cells giving rise to smaller conidia. **i** Conjugation tube formation between two conidia. **j** Conidium disintegrating on MEA. Bars: B-C, F-I = 10 μm; E, J = 5 μm; D = 2,5 μm

MycoBank MB 826878. (Fig. 6)

*Etymology*: The epithet refers to the variable size and shape of the conidia.

*Diagnosis*: *Lecanosticta variabilis* is distinguished from the closely related species, *L. acicola*, by either ITS, *TEF*1 or *MS204*. Morphologically, it is distinguished from other species with the exception of *L. acicola* by the diffusion of sulphur-yellow to cinnamon metabolite into PDA and a luteus to sienna coloured metabolite produced on MEA within 2 wk. This species also has smaller conidia than those of *L. acicola*.

*Type*: **Honduras**: Santa Barbara, on needles of *Pinus caribaea*, 1980, *H.C. Evans*, (PREM 62196 – holotype; CMW 42205 = IMI 281561 = CBS 144453 – ex-type culture).

*Description*: *Sexual state* not observed. *Conidiomata* olivaceous to vinaceous brown on MEA. *Conidiophores* cylindrical, extending in densely aggregated palisade, hyaline to honey to pale vinaceous brown, smooth to verruculose, unbranched or branched at base, septate or aseptate, often encased in granular yellow to light brown mucoid sheath, length up to 60 μm, 2.0–5.0 μm diam. *Conidiogenous cells* terminal, integrated, subcylindrical to cylindrical, hyaline to light brown, smooth to verruculose, proliferating several times percurrently with visible annelations near apex, septate or aseptate, (4.5–)5.5–10.5(− 12.0) × (1.5–)2.0–3.5(− 5.0) μm. *Conidia* three different conidial types. All three types solitary, smooth to verruculose, subhyaline to honey to light brown, often enclosed in granular light luteus mucoid sheath. Type 1 straight to strongly curved, subcylindrical to cylindrical, subobtusely rounded apex, truncate, 1–4-septate, base (1.5–)2.0–2.5(− 3.0) μm diam. (22–)25.0–34.0(− 43.0) × (2.0–)2.5–3.0(− 3.5) μm. Type 2 slightly curved, cylindrical with both apex and base rounded, 0–2-septate, (14.5–)15.5–19.5(− 22.0) × (2.0–)2.5–3.0(− 3.5) μm. Type 3 buds from larger conidia (see notes) or from conidiogenous cells, hyaline, fusiform to cylindrical with subobtusely rounded apex and base, 0–1-septate, (10.0–)11.0–14.0(− 15.5) × (2.0–)2.0–2.5(− 3.0) μm.

*Culture characteristics*: Colonies flat to somewhat erumpent, spreading, with sparse aerial mycelium, surface folded, with smooth, lobate margins. On MEA, surface isabelline with patches of pale luteus to dark olivaceous green, reverse olivaceous to fuscous black. Mucoid yellow to peach to yellow-green exudate present. Luteus to sienna coloured metabolite diffusing into medium. On PDA, surface isabelline in centre, rosy buff in outer region, dark olivacous-brown on edges and isabelline in reverse. Sulphur yellow to cinnamon coloured metabolite diffuses into media. On OA, surface dirty white with diffuse umber outer region. *Growth characteristics*: optimal growth temperature 25 °C, after 4 wk., colonies at 10, 15, 20, 25 and 30 °C reached maximum of 11.5, 21, 31, 31.5 and 22.5 mm, with mean growth of 2.2, 4.5, 6.1, 6.9 and 3.6 mm / wk. respectively.

*Notes*: The cells in the conidia often swell and break off, forming endospores as described in *L. acicola* (Siggers 1950; Crosby 1966; Evans 1984). Secondary conidia were commonly produced in cultures of this species, similar to those previously described for *L. acicola* specimens examined directly from needles (Evans 1984).

*Additional material examined*: **Guatemala**: Alta Verapaz, Santa Cruz Verapaz, near Tactíc, on needles of *Pinus oocarpa*, 21 Oct 2010, *I. Barnes* (PREM 62194, CMW 37125 = CBS 144454 – culture); *loc. cit., I. Barnes* (culture CMW 37129); Jalapa, Finca Forestal Soledad, on needles of *Pinus maximinoi*, 21 Oct 2010, *I. Barnes* (PREM 62195, CMW 36809 = CBS 144455 – culture). **–Mexico**: on needles of a *Pinus* sp., 30 Nov 2009, *M. de Jesús Yáñez-Morales* (CBS H-21112; culture CMW45425 = CPC 17822 = CBS 133789);

## DISCUSSION

Four novel species of *Lecanostica* from infected pine needles collected in Central America are reported and named as *L. jani*, *L. pharomachri*, *L. tecunumanii*, and *L. variabilis*. There are now nine species described in the genus and these can be distinguished based on a phylogenetic inference for multiple gene regions. The two previously described species, *L. brevispora* and *L. guatemalensis,* were also found in this study and they provide new host and country records. The well-known pine pathogen, *L. acicola*, was not found on any of the samples collected from five *Pinus* spp*.* in seven regions of Central America considered in this study. This suggests that the species is not native in that region.

Results of the present study support the view of Quaedvlieg et al. (2012) that a combination of the ITS and *TEF*1 should be used as barcoding loci to distinguish between species of *Lecanosticta* and other closely related species. Additionally, statistically well supported clades were obtained in this study using the *MS204* gene region. However, genus-specific primers should ideally be designed to increase the amplification success rate for this gene region in *Lecanosticta*. Although the *BT*2 gene was also proposed as a possible barcoding region that could be used to distinguish between *Lecanosticta* species and other species of *Mycosphaerellaceae* (Quaedvlieg et al. 2012), it amplified poorly in the present study. The *BT*1 gene region distinguished most of the species, but not *L. pharomachri* and *L. variabilis* and provided low statistical support at all nodes.

The results of this study support the view of Evans (1984) that *Lecanostica* species are comprised of morphotypes or ecotypes. Based on phylogenetic analyses, we were able to define lineages for species also supported by morphological characteristics. The *TEF*1 sequences were highly variable but several well supported clades and subclades were observed within species (Fig. 2). These clades possibly represent additional new species but we lacked sufficient cultures and support to describe them. The clade with the most diversity in terms of unique *TEF*1 haplotypes, Clade 1, was *L. brevispora* (represented by 22.1% of *TEF*1 haplotypes in the genus) and this species was also represented by the largest number of isolates. High haplotype diversity was observed in the *L. jani* (16.1% of *TEF*1 haplotypes) and *L. pharomachri* (10.3% of *TEF*1 haplotypes) clades and different lineages were observed in the *L. acicola* (13.2% of *TEF*1 haplotypes)*, L. guatemalensis* (17.6% of *TEF*1 haplotypes), and *L. variabilis* (13.2% of *TEF*1 haplotypes) clades. The other gene regions, especially *MS204* and *RPB*2 were also highly variable in terms of distinguishing haplotypes. *RPB*2 is however, not recommended to distinguish between *L. acicola* and *L. variabilis* as these two species form paraphyletic groups in the tree for this gene region.

The paleo-geographic region that includes Mexico and extends into Central America is regarded as one of three centres of diversity of *Pinus* species (Farjon 1996). Pine needles that were sampled from Central America in this study were symptomatic but serious disease was not observed. This suggests that *Lecanosticta* species have co-speciated with their native pine hosts in this region. Of the nine known species, *L. gloeospora* and *L. longispora* have been identified only in Mexico and *L. brevispora* and *L. variabilis* have been identified in both Mexico and Central America. *Lecanosticta guatemalensis, L. jani, L. pharomachri* and *L. tecunumanii* are currently known only from Central America.

*Lecanosticta acicola* has been redefined in this study. All isolates from Central America that had previously been identified as *L. acicola,* based on morphological characteristics, are now treated as different species. This is based on newly available DNA sequence data and phylogenetic analyses emerging from this study as well as that of Quaedvlieg et al. (2012). *L. acicola* is, however, still considered as present in Mexico.

Based on *TEF*1 analyses, *L. acicola* resolves in three lineages. Janoušek et al. (2016) used microsatellites to show that a lineage of *L. acicola* from the northern USA was introduced into Central and Northern Europe, and a lineage from the southern USA was introduced into France, Spain, and Colombia. Similarly, Huang et al. (1995) reported that *L. acicola* was introduced into China from the southern part of the USA. Our analyses of the *TEF*1 sequences of isolates from the northern parts of the USA, Lithuania, and a representative sequence for Central and Northern Europe and Canada (KJ938438, Table 3), formed one distinct lineage with *L. acicola* (Fig. 2). All isolates from the southern parts of the USA, as well as representative sequences for Asia, France, Spain, and Colombia (Table 3), formed a second distinct lineage in the clade accommodating *L. acicola* (Fig. 2). The third lineage included only isolates from Mexico, which suggests that isolates in this lineage have remained in their area of origin and have not been introduced elsewhere. Because this Mexican lineage had strong bootstrap support separating it from the other two lineages, it could represent a further new species. Only *TEF*1 data are currently available for the Mexican collections (downloaded from GenBank) and other gene regions would need to be sequenced and analysed to determine whether this really represents a further novel taxon.

Evans (1984) first speculated that Central America could be the centre of origin of *Lecanosticta*. The phylogenetic analyses conducted in the present study showed that there is a high diversity of species and lineages for this genus in Central America, which supports Evans’ hypothesis. This is the first study where all known species of *Lecanosticta* have been delineated based on DNA sequence data and phylogenetic analysis, and it has led to the recognition of additional new taxa from Central America and Mexico. Eight of the nine species of *Lecanosticta* have been reported only from this region, and our results consequently represent strong support for a Mesoamerican *Lecanosticta* centre of diversity and likely origin. Population genetic analyses for the most common of these species will serve to provide additional support for this hypothesis.

## CONCLUSIONS

Phylogenetic inference based on DNA sequence data including new collections from Mexico and Central America revealed four novel species and reaffirmed the identity of the five previously described taxa. The most important of these species is the well-known pine pathogen *L. acicola* that was redefined as a North American taxon and for which at least three distinct lineages can be distinguished using the *TEF*1 gene region. New regions of occurrence and host range emerged for *Lecanosticta* spp. with eight of the nine species occurring in Mesoamerica. This suggests that Mesoamerica is the most likely centre of origin for *Lecanosticta*. *Lecanosticta acicola* was best known as the causal agent of the important brown spot needle blight of *Pinus palustris* in the southeastern USA but it has more recently spread within the USA and Europe where it has become an increasingly important pathogen of numerous *Pinus* spp. The other species of *Lecanosticta,* including those newly described, are of unknown importance but it seems likely that some of them could pose a threat to *Pinus* spp. if they were introduced into new environments in the future. The fact that various Mesoamerican *Pinus* spp. are increasingly being used for plantation development in the Southern Hemisphere implies that extreme caution should be applied not to introduce *Lecanosticta* spp. together with germplasm needed for future planting programmes.

## Additional files

Additional file 1: **Figure S1.** Maximum likelihood tree representing the five known and four novel species of *Lecanosticta* generated from the ITS region. MP bootstrap support (> 70%) are indicated first, followed by ML bootstrap values (MP/ML, * = insignificant value). Bold branches indicate BI values > than 0.95. *Dothistroma* species were used as the outgroup taxa. All represented type species are indicated in bold and with a “T”. Clades indicated on the left correspond with the clades in Fig. 1. Within the *L. jani* clade a “∆” next to the isolate indicates that the isolate exhibits Type 2 morphology but it groups with Subclade 1 or exhibits Type 1 morphology but groups with Subclade 2. (PPTX 61 kb)

Additional file 2: **Figure S2.** Maximum likelihood tree representing the five known and four novel species of *Lecanosticta* generated from the *BT*1 region. MP bootstrap support (> 70%) are indicated first, followed by ML bootstrap values (MP/ML, * = insignificant value). Bold branches indicate BI values > than 0.95. *Dothistroma* species were used as the outgroup taxa. All represented type species are indicated in bold and with a “T”. Clades indicated on the left correspond with the clades in Fig. 1. (PPTX 54 kb)

Additional file 3: **Figure S3.** Maximum likelihood tree representing the five known and four novel species of *Lecanosticta* generated from the *MS204* region. MP bootstrap support (> 70%) are indicated first, followed by ML bootstrap values (MP/ML, * = insignificant value). Bold branches indicate BI values > than 0.95. *Dothistroma septosporum* was used as the outgroup taxa. All represented type species are indicated in bold and with a “T”. Clades indicated on the left correspond with the clades in Fig. 1. Within the *L. jani* clade a “∆” next to the isolate indicates that the isolate exhibits Type 2 morphology but it groups with Subclade 1 or exhibits Type 1 morphology but groups with Subclade 2. (PPTX 55 kb)

Additional file 4: **Figure S4.** Maximum likelihood tree representing the five known and four novel species of *Lecanosticta* generated from the *RPB*2 region. MP bootstrap support (> 70%) are indicated first, followed by ML bootstrap values (MP/ML, * = insignificant value). Bold branches indicate BI values > than 0.95. *Dothistroma* species were used as the outgroup taxa. All represented type species are indicated in bold and with a “T”. Clades indicated on the left correspond with the clades in Fig. 1. Within the *L. jani* clade a “∆” next to the isolate indicates that the isolate exhibits Type 2 morphology but it groups with Subclade 1 or exhibits Type 1 morphology but groups with Subclade 2. (PPTX 61 kb)

## Abbreviations

1F1N: One Fungus One Name
AIC: Akaike Information Criterion
BI: Bayesian inference
BSNB: Brown spot needle blight
*BT*1: Beta-tubulin-1 gene region
*BT*2: Beta-tubulin-2 gene region
CA: California
CBS: The culture collection of the Westerdijk Fungal Biodiversity Institute, Utrecht, The Netherlands
CI: Consistency index
CMW: The culture collection of the Forestry and Agricultural Biotechnology Institute
COSAVE: El Comité de Sanidad Vegetal
CPC: Personal collection of Pedro Crous housed at CBS
DSM: Dothistroma Sporulating Media
FABI: Forestry and Agricultural Biotechnology Institute
HI: Homoplasy index
IASPC: Inter-African Phytosanitary Council
ICN: International Code of Nomenclature for algae, fungi, and plants
IMI: The UK National Fungus Collection maintained by CABI Bioscience, Egham, UK
ITS: Internal transcribed spacers
MA: Massachusetts
MB: MycoBank
MCMC: Markov Chain Monte Carlo
MD: Maryland
ME: Maine
MEA: Malt Extract Agar
ML: Maximum likelihood
MO: Missouri
MP: Maximum parsimony
*MS204*: The guanine nucleotide-binding protein subunit beta
NCBI: National Centre for Biotechnology Information
NJ: New Jersey
OA: Oatmeal Agar
PCR: Polymerase chain reaction
PDA: Potato Dextrose Agar
PHT: Partition homogeneity test
PREM: The dried herbarium collection of the South African National Collection of Fungi
RC: Rescaled consistency index
RI: Retention index
*RPB*2: RNA polymerase II second largest subunit
TBR: Tree-bisection-reconnection
*TEF*1: Translation elongation factor 1-α gene
TL: Tree length
