# Supplementary material for: Biodiversity of Lecanosticta pine-needle blight pathogens suggests a Mesoamerican Centre of origin
Source: IMA Fungus. 2019 Jun 7;10:2. doi: 10.1186/s43008-019-0004-8 (PMC7325671; doi:10.1186/s43008-019-0004-8)
Supplement: Supplementary file 1 — Figure S1. Maximum likelihood tree representing the five known and four novel species of Lecanosticta generated from the ITS region. MP bootstrap support (> 70%) are indicated first, followed by ML bootstrap values (MP/ML, * = insignificant value). Bold branches indicate BI values > than 0.95. Dothistroma species were used as the outgroup taxa. All represented type species are indicated in bold and with a “T”. Clades indicated on the left correspond with the clades in Fig. 1. Within the L. jani clade a “∆” next to the isolate indicates that the isolate exhibits Type 2 morphology but it groups with Subclade 1 or exhibits Type 1 morphology but groups with Subclade 2. (PPTX 61 kb) [file 43008_2019_4_MOESM1_ESM.pptx]

## Slide 1
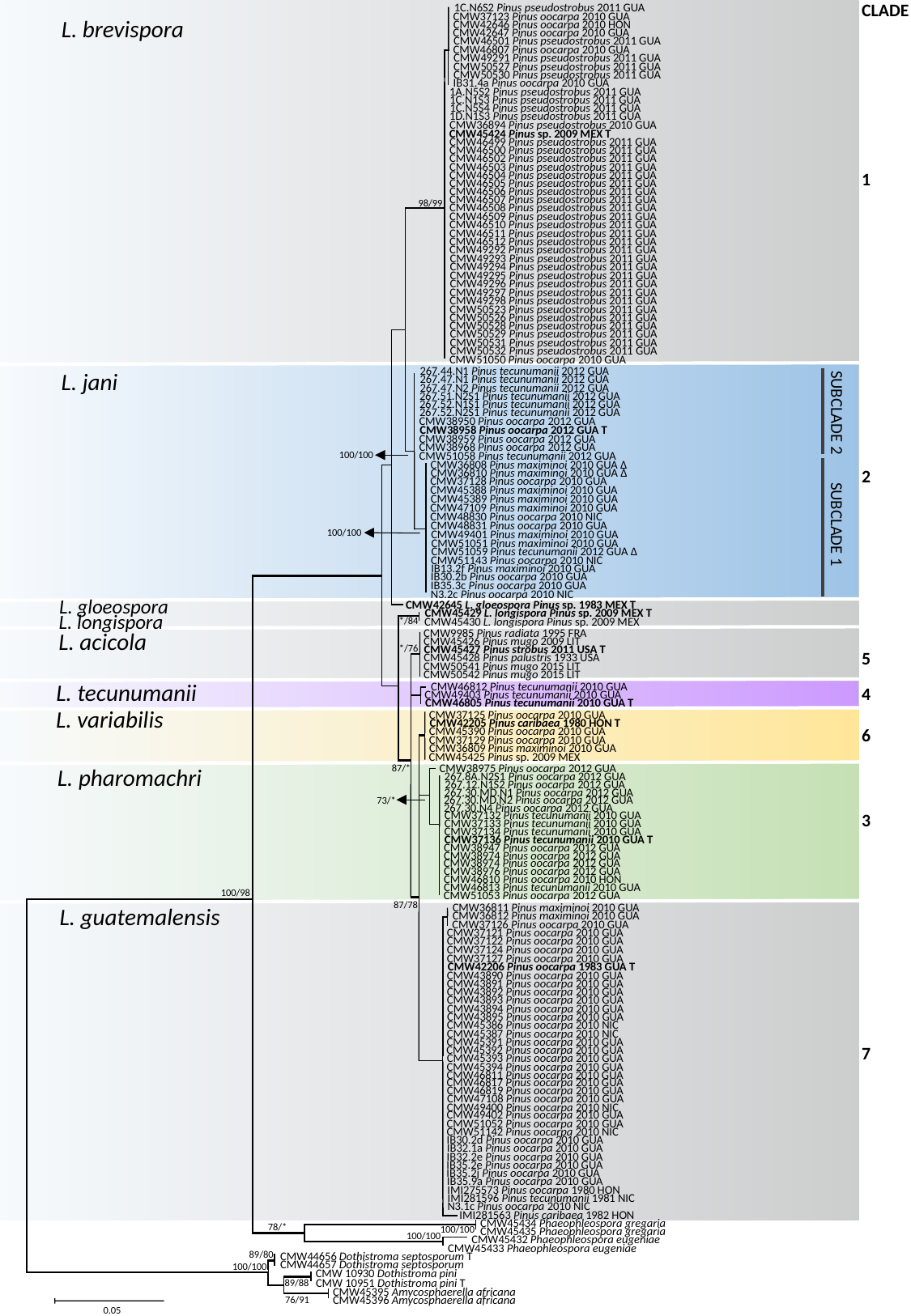

CLADE
1
2
5
4
6
3
7
 1C.N6S2 Pinus pseudostrobus 2011 GUA
L. brevispora
 CMW37123 Pinus oocarpa 2010 GUA
 CMW42646 Pinus oocarpa 2010 HON
 CMW42647 Pinus oocarpa 2010 GUA
 CMW46501 Pinus pseudostrobus 2011 GUA
 CMW46807 Pinus oocarpa 2010 GUA
 CMW49291 Pinus pseudostrobus 2011 GUA
 CMW50527 Pinus pseudostrobus 2011 GUA
 CMW50530 Pinus pseudostrobus 2011 GUA
 IB31.4a Pinus oocarpa 2010 GUA
 1A.N5S2 Pinus pseudostrobus 2011 GUA
 1C.N1S3 Pinus pseudostrobus 2011 GUA
 1C.N5S4 Pinus pseudostrobus 2011 GUA
 1D.N1S3 Pinus pseudostrobus 2011 GUA
 CMW36894 Pinus pseudostrobus 2010 GUA
 CMW45424 Pinus sp. 2009 MEX T
 CMW46499 Pinus pseudostrobus 2011 GUA
 CMW46500 Pinus pseudostrobus 2011 GUA
 CMW46502 Pinus pseudostrobus 2011 GUA
 CMW46503 Pinus pseudostrobus 2011 GUA
 CMW46504 Pinus pseudostrobus 2011 GUA
 CMW46505 Pinus pseudostrobus 2011 GUA
 CMW46506 Pinus pseudostrobus 2011 GUA
98/99
 CMW46507 Pinus pseudostrobus 2011 GUA
 CMW46508 Pinus pseudostrobus 2011 GUA
 CMW46509 Pinus pseudostrobus 2011 GUA
 CMW46510 Pinus pseudostrobus 2011 GUA
 CMW46511 Pinus pseudostrobus 2011 GUA
 CMW46512 Pinus pseudostrobus 2011 GUA
 CMW49292 Pinus pseudostrobus 2011 GUA
 CMW49293 Pinus pseudostrobus 2011 GUA
 CMW49294 Pinus pseudostrobus 2011 GUA
 CMW49295 Pinus pseudostrobus 2011 GUA
 CMW49296 Pinus pseudostrobus 2011 GUA
 CMW49297 Pinus pseudostrobus 2011 GUA
 CMW49298 Pinus pseudostrobus 2011 GUA
 CMW50523 Pinus pseudostrobus 2011 GUA
 CMW50526 Pinus pseudostrobus 2011 GUA
 CMW50528 Pinus pseudostrobus 2011 GUA
 CMW50529 Pinus pseudostrobus 2011 GUA
 CMW50531 Pinus pseudostrobus 2011 GUA
 CMW50532 Pinus pseudostrobus 2011 GUA
 CMW51050 Pinus oocarpa 2010 GUA
L. jani
 267.44.N1 Pinus tecunumanii 2012 GUA
 267.47.N1 Pinus tecunumanii 2012 GUA
 267.47.N2 Pinus tecunumanii 2012 GUA
 267.51.N2S1 Pinus tecunumanii 2012 GUA
SUBCLADE 2
 267.52.N1S1 Pinus tecunumanii 2012 GUA
 267.52.N2S1 Pinus tecunumanii 2012 GUA
 CMW38950 Pinus oocarpa 2012 GUA
 CMW38958 Pinus oocarpa 2012 GUA T
 CMW38959 Pinus oocarpa 2012 GUA
 CMW38968 Pinus oocarpa 2012 GUA
100/100
 CMW51058 Pinus tecunumanii 2012 GUA
 CMW36808 Pinus maximinoi 2010 GUA ∆
 CMW36810 Pinus maximinoi 2010 GUA ∆
 CMW37128 Pinus oocarpa 2010 GUA
 CMW45388 Pinus maximinoi 2010 GUA
 CMW45389 Pinus maximinoi 2010 GUA
 CMW47109 Pinus maximinoi 2010 GUA
SUBCLADE 1
 CMW48830 Pinus oocarpa 2010 NIC
 CMW48831 Pinus oocarpa 2010 GUA
100/100
 CMW49401 Pinus maximinoi 2010 GUA
 CMW51051 Pinus maximinoi 2010 GUA
 CMW51059 Pinus tecunumanii 2012 GUA ∆
 CMW51143 Pinus oocarpa 2010 NIC
 IB13.2f Pinus maximinoi 2010 GUA
 IB30.2b Pinus oocarpa 2010 GUA
 IB35.3c Pinus oocarpa 2010 GUA
 N3.2c Pinus oocarpa 2010 NIC
L. gloeospora
CMW42645 L. gloeospora Pinus sp. 1983 MEX T
L. longispora
 CMW45429 L. longispora Pinus sp. 2009 MEX T
*/84
 CMW45430 L. longispora Pinus sp. 2009 MEX
L. acicola
 CMW9985 Pinus radiata 1995 FRA
 CMW45426 Pinus mugo 2009 LIT
*/76
 CMW45427 Pinus strobus 2011 USA T
 CMW45428 Pinus palustris 1933 USA
 CMW50541 Pinus mugo 2015 LIT
 CMW50542 Pinus mugo 2015 LIT
L. tecunumanii
 CMW46812 Pinus tecunumanii 2010 GUA
 CMW49403 Pinus tecunumanii 2010 GUA
 CMW46805 Pinus tecunumanii 2010 GUA T
L. variabilis
 CMW37125 Pinus oocarpa 2010 GUA
 CMW42205 Pinus caribaea 1980 HON T
 CMW45390 Pinus oocarpa 2010 GUA
 CMW37129 Pinus oocarpa 2010 GUA
 CMW36809 Pinus maximinoi 2010 GUA
 CMW45425 Pinus sp. 2009 MEX
87/*
L. pharomachri
 CMW38975 Pinus oocarpa 2012 GUA
 267.8A.N2S1 Pinus oocarpa 2012 GUA
 267.12.N1S2 Pinus oocarpa 2012 GUA
 267.30.MD.N1 Pinus oocarpa 2012 GUA
73/*
 267.30.MD.N2 Pinus oocarpa 2012 GUA
 267.30.N4 Pinus oocarpa 2012 GUA
 CMW37132 Pinus tecunumanii 2010 GUA
 CMW37133 Pinus tecunumanii 2010 GUA
 CMW37134 Pinus tecunumanii 2010 GUA
 CMW37136 Pinus tecunumanii 2010 GUA T
 CMW38947 Pinus oocarpa 2012 GUA
 CMW38974 Pinus oocarpa 2012 GUA
 CMW38974 Pinus oocarpa 2012 GUA
 CMW38976 Pinus oocarpa 2012 GUA
 CMW46810 Pinus oocarpa 2010 HON
 CMW46813 Pinus tecunumanii 2010 GUA
100/98
 CMW51053 Pinus oocarpa 2012 GUA
87/78
L. guatemalensis
 CMW36811 Pinus maximinoi 2010 GUA
 CMW36812 Pinus maximinoi 2010 GUA
 CMW37126 Pinus oocarpa 2010 GUA
 CMW37121 Pinus oocarpa 2010 GUA
 CMW37122 Pinus oocarpa 2010 GUA
 CMW37124 Pinus oocarpa 2010 GUA
 CMW37127 Pinus oocarpa 2010 GUA
 CMW42206 Pinus oocarpa 1983 GUA T
 CMW43890 Pinus oocarpa 2010 GUA
 CMW43891 Pinus oocarpa 2010 GUA
 CMW43892 Pinus oocarpa 2010 GUA
 CMW43893 Pinus oocarpa 2010 GUA
 CMW43894 Pinus oocarpa 2010 GUA
 CMW43895 Pinus oocarpa 2010 GUA
 CMW45386 Pinus oocarpa 2010 NIC
 CMW45387 Pinus oocarpa 2010 NIC
 CMW45391 Pinus oocarpa 2010 GUA
 CMW45392 Pinus oocarpa 2010 GUA
 CMW45393 Pinus oocarpa 2010 GUA
 CMW45394 Pinus oocarpa 2010 GUA
 CMW46811 Pinus oocarpa 2010 GUA
 CMW46817 Pinus oocarpa 2010 GUA
 CMW46819 Pinus oocarpa 2010 GUA
 CMW47108 Pinus oocarpa 2010 GUA
 CMW49400 Pinus oocarpa 2010 NIC
 CMW49402 Pinus oocarpa 2010 GUA
 CMW51052 Pinus oocarpa 2010 GUA
 CMW51142 Pinus oocarpa 2010 NIC
 IB30.2d Pinus oocarpa 2010 GUA
 IB32.1a Pinus oocarpa 2010 GUA
 IB32.2e Pinus oocarpa 2010 GUA
 IB35.2e Pinus oocarpa 2010 GUA
 IB35.2j Pinus oocarpa 2010 GUA
 IB35.9a Pinus oocarpa 2010 GUA
 IMI275573 Pinus oocarpa 1980 HON
 IMI281596 Pinus tecunumanii 1981 NIC
 N3.1c Pinus oocarpa 2010 NIC
 IMI281563 Pinus caribaea 1982 HON
78/*
 CMW45434 Phaeophleospora gregaria
100/100
100/100
 CMW45435 Phaeophleospora gregaria
 CMW45432 Phaeophleospora eugeniae
 CMW45433 Phaeophleospora eugeniae
89/80
 CMW44656 Dothistroma septosporum T
100/100
 CMW44657 Dothistroma septosporum
 CMW 10930 Dothistroma pini
89/88
 CMW 10951 Dothistroma pini T
 CMW45395 Amycosphaerella africana
76/91
 CMW45396 Amycosphaerella africana
0.05
