# Supplementary material for: Biodiversity of Lecanosticta pine-needle blight pathogens suggests a Mesoamerican Centre of origin
Source: IMA Fungus. 2019 Jun 7;10:2. doi: 10.1186/s43008-019-0004-8 (PMC7325671; doi:10.1186/s43008-019-0004-8)
Supplement: Supplementary file 2 — Figure S2. Maximum likelihood tree representing the five known and four novel species of Lecanosticta generated from the BT1 region. MP bootstrap support (> 70%) are indicated first, followed by ML bootstrap values (MP/ML, * = insignificant value). Bold branches indicate BI values > than 0.95. Dothistroma species were used as the outgroup taxa. All represented type species are indicated in bold and with a “T”. Clades indicated on the left correspond with the clades in Fig. 1. (PPTX 54 kb) [file 43008_2019_4_MOESM2_ESM.pptx]

## Slide 1
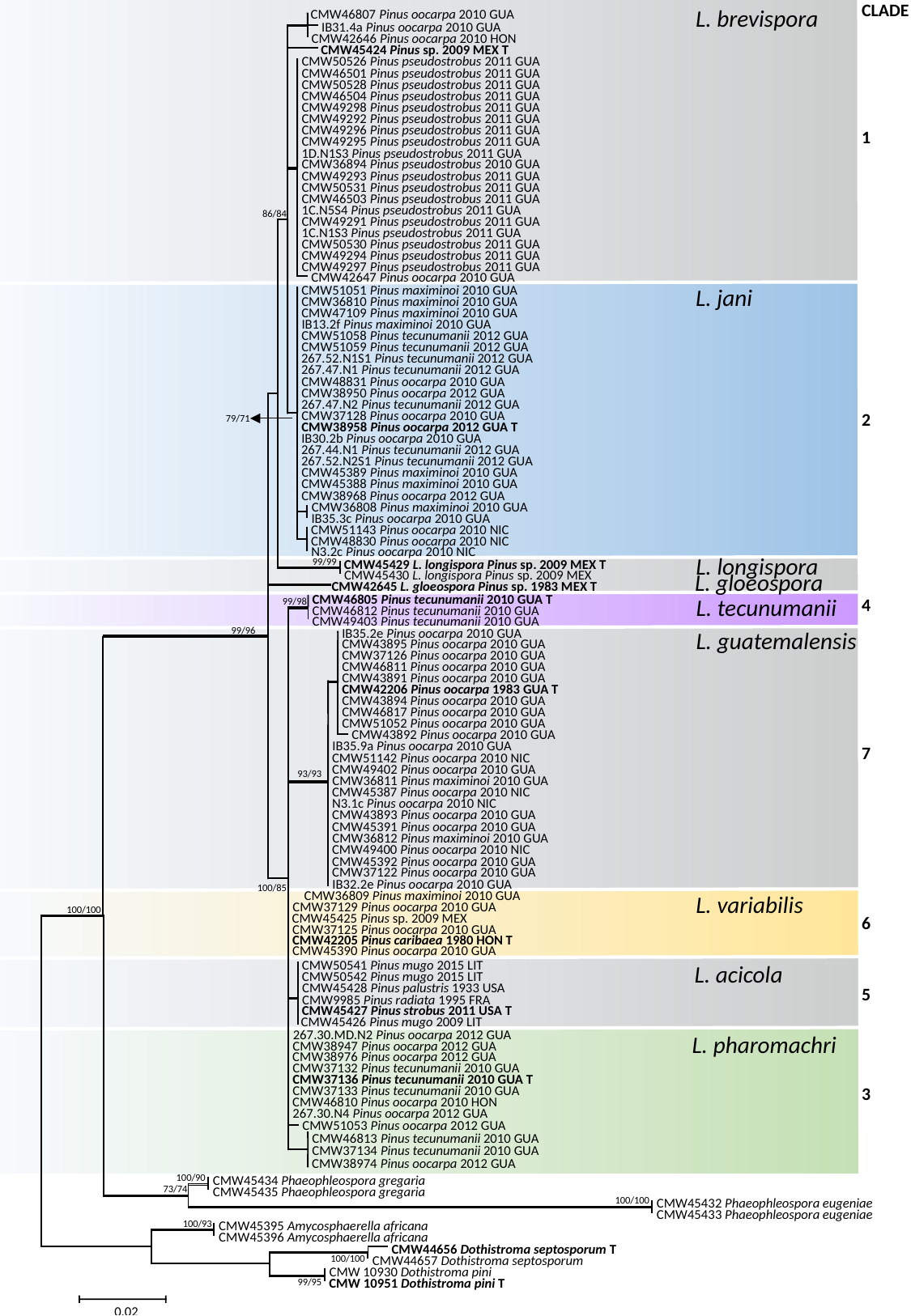

CLADE
1
2
4
7
6
5
3
L. brevispora
CMW46807 Pinus oocarpa 2010 GUA
 IB31.4a Pinus oocarpa 2010 GUA
 CMW42646 Pinus oocarpa 2010 HON
 CMW45424 Pinus sp. 2009 MEX T
 CMW50526 Pinus pseudostrobus 2011 GUA
 CMW46501 Pinus pseudostrobus 2011 GUA
 CMW50528 Pinus pseudostrobus 2011 GUA
 CMW46504 Pinus pseudostrobus 2011 GUA
 CMW49298 Pinus pseudostrobus 2011 GUA
 CMW49292 Pinus pseudostrobus 2011 GUA
 CMW49296 Pinus pseudostrobus 2011 GUA
 CMW49295 Pinus pseudostrobus 2011 GUA
 1D.N1S3 Pinus pseudostrobus 2011 GUA
 CMW36894 Pinus pseudostrobus 2010 GUA
 CMW49293 Pinus pseudostrobus 2011 GUA
 CMW50531 Pinus pseudostrobus 2011 GUA
 CMW46503 Pinus pseudostrobus 2011 GUA
86/84
 1C.N5S4 Pinus pseudostrobus 2011 GUA
 CMW49291 Pinus pseudostrobus 2011 GUA
 1C.N1S3 Pinus pseudostrobus 2011 GUA
 CMW50530 Pinus pseudostrobus 2011 GUA
 CMW49294 Pinus pseudostrobus 2011 GUA
 CMW49297 Pinus pseudostrobus 2011 GUA
 CMW42647 Pinus oocarpa 2010 GUA
L. jani
 CMW51051 Pinus maximinoi 2010 GUA
 CMW36810 Pinus maximinoi 2010 GUA
 CMW47109 Pinus maximinoi 2010 GUA
 IB13.2f Pinus maximinoi 2010 GUA
 CMW51058 Pinus tecunumanii 2012 GUA
 CMW51059 Pinus tecunumanii 2012 GUA
 267.52.N1S1 Pinus tecunumanii 2012 GUA
 267.47.N1 Pinus tecunumanii 2012 GUA
 CMW48831 Pinus oocarpa 2010 GUA
 CMW38950 Pinus oocarpa 2012 GUA
 267.47.N2 Pinus tecunumanii 2012 GUA
79/71
 CMW37128 Pinus oocarpa 2010 GUA
 CMW38958 Pinus oocarpa 2012 GUA T
 IB30.2b Pinus oocarpa 2010 GUA
 267.44.N1 Pinus tecunumanii 2012 GUA
 267.52.N2S1 Pinus tecunumanii 2012 GUA
 CMW45389 Pinus maximinoi 2010 GUA
 CMW45388 Pinus maximinoi 2010 GUA
 CMW38968 Pinus oocarpa 2012 GUA
 CMW36808 Pinus maximinoi 2010 GUA
 IB35.3c Pinus oocarpa 2010 GUA
 CMW51143 Pinus oocarpa 2010 NIC
 CMW48830 Pinus oocarpa 2010 NIC
 N3.2c Pinus oocarpa 2010 NIC
L. longispora
99/99
 CMW45429 L. longispora Pinus sp. 2009 MEX T
L. gloeospora
 CMW45430 L. longispora Pinus sp. 2009 MEX
CMW42645 L. gloeospora Pinus sp. 1983 MEX T
L. tecunumanii
99/98
 CMW46805 Pinus tecunumanii 2010 GUA T
 CMW46812 Pinus tecunumanii 2010 GUA
 CMW49403 Pinus tecunumanii 2010 GUA
99/96
L. guatemalensis
 IB35.2e Pinus oocarpa 2010 GUA
 CMW43895 Pinus oocarpa 2010 GUA
 CMW37126 Pinus oocarpa 2010 GUA
 CMW46811 Pinus oocarpa 2010 GUA
 CMW43891 Pinus oocarpa 2010 GUA
 CMW42206 Pinus oocarpa 1983 GUA T
 CMW43894 Pinus oocarpa 2010 GUA
 CMW46817 Pinus oocarpa 2010 GUA
 CMW51052 Pinus oocarpa 2010 GUA
 CMW43892 Pinus oocarpa 2010 GUA
 IB35.9a Pinus oocarpa 2010 GUA
 CMW51142 Pinus oocarpa 2010 NIC
 CMW49402 Pinus oocarpa 2010 GUA
93/93
 CMW36811 Pinus maximinoi 2010 GUA
 CMW45387 Pinus oocarpa 2010 NIC
 N3.1c Pinus oocarpa 2010 NIC
 CMW43893 Pinus oocarpa 2010 GUA
 CMW45391 Pinus oocarpa 2010 GUA
 CMW36812 Pinus maximinoi 2010 GUA
 CMW49400 Pinus oocarpa 2010 NIC
 CMW45392 Pinus oocarpa 2010 GUA
 CMW37122 Pinus oocarpa 2010 GUA
 IB32.2e Pinus oocarpa 2010 GUA
100/85
L. variabilis
 CMW36809 Pinus maximinoi 2010 GUA
100/100
 CMW37129 Pinus oocarpa 2010 GUA
 CMW45425 Pinus sp. 2009 MEX
 CMW37125 Pinus oocarpa 2010 GUA
 CMW42205 Pinus caribaea 1980 HON T
 CMW45390 Pinus oocarpa 2010 GUA
L. acicola
 CMW50541 Pinus mugo 2015 LIT
 CMW50542 Pinus mugo 2015 LIT
 CMW45428 Pinus palustris 1933 USA
 CMW9985 Pinus radiata 1995 FRA
 CMW45427 Pinus strobus 2011 USA T
 CMW45426 Pinus mugo 2009 LIT
L. pharomachri
 267.30.MD.N2 Pinus oocarpa 2012 GUA
 CMW38947 Pinus oocarpa 2012 GUA
 CMW38976 Pinus oocarpa 2012 GUA
 CMW37132 Pinus tecunumanii 2010 GUA
 CMW37136 Pinus tecunumanii 2010 GUA T
 CMW37133 Pinus tecunumanii 2010 GUA
 CMW46810 Pinus oocarpa 2010 HON
 267.30.N4 Pinus oocarpa 2012 GUA
 CMW51053 Pinus oocarpa 2012 GUA
 CMW46813 Pinus tecunumanii 2010 GUA
 CMW37134 Pinus tecunumanii 2010 GUA
 CMW38974 Pinus oocarpa 2012 GUA
100/90
 CMW45434 Phaeophleospora gregaria
73/74
 CMW45435 Phaeophleospora gregaria
100/100
 CMW45432 Phaeophleospora eugeniae
 CMW45433 Phaeophleospora eugeniae
100/93
 CMW45395 Amycosphaerella africana
 CMW45396 Amycosphaerella africana
 CMW44656 Dothistroma septosporum T
100/100
 CMW44657 Dothistroma septosporum
 CMW 10930 Dothistroma pini
99/95
 CMW 10951 Dothistroma pini T
0.02
