# Supplementary material for: Biodiversity of Lecanosticta pine-needle blight pathogens suggests a Mesoamerican Centre of origin
Source: IMA Fungus. 2019 Jun 7;10:2. doi: 10.1186/s43008-019-0004-8 (PMC7325671; doi:10.1186/s43008-019-0004-8)
Supplement: Supplementary file 3 — Figure S3. Maximum likelihood tree representing the five known and four novel species of Lecanosticta generated from the MS204 region. MP bootstrap support (> 70%) are indicated first, followed by ML bootstrap values (MP/ML, * = insignificant value). Bold branches indicate BI values > than 0.95. Dothistroma septosporum was used as the outgroup taxa. All represented type species are indicated in bold and with a “T”. Clades indicated on the left correspond with the clades in Fig. 1. Within the L. jani clade a “∆” next to the isolate indicates that the isolate exhibits Type 2 morphology but it groups with Subclade 1 or exhibits Type 1 morphology but groups with Subclade 2. (PPTX 55 kb) [file 43008_2019_4_MOESM3_ESM.pptx]

## Slide 1
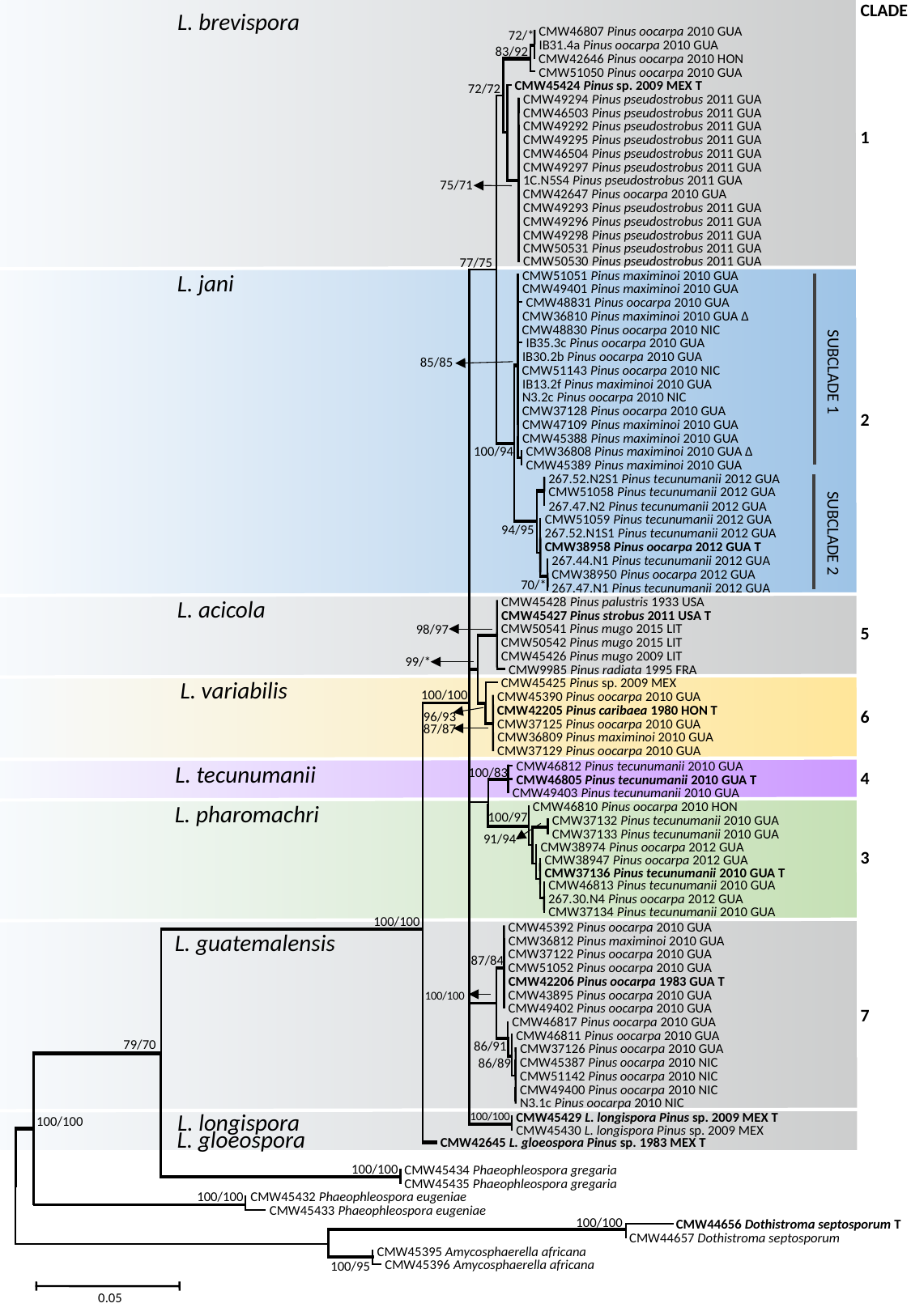

CLADE
1
2
5
6
4
3
7
L. brevispora
72/*
 CMW46807 Pinus oocarpa 2010 GUA
 IB31.4a Pinus oocarpa 2010 GUA
83/92
 CMW42646 Pinus oocarpa 2010 HON
 CMW51050 Pinus oocarpa 2010 GUA
72/72
 CMW45424 Pinus sp. 2009 MEX T
 CMW49294 Pinus pseudostrobus 2011 GUA
 CMW46503 Pinus pseudostrobus 2011 GUA
 CMW49292 Pinus pseudostrobus 2011 GUA
 CMW49295 Pinus pseudostrobus 2011 GUA
 CMW46504 Pinus pseudostrobus 2011 GUA
 CMW49297 Pinus pseudostrobus 2011 GUA
75/71
 1C.N5S4 Pinus pseudostrobus 2011 GUA
 CMW42647 Pinus oocarpa 2010 GUA
 CMW49293 Pinus pseudostrobus 2011 GUA
 CMW49296 Pinus pseudostrobus 2011 GUA
 CMW49298 Pinus pseudostrobus 2011 GUA
 CMW50531 Pinus pseudostrobus 2011 GUA
77/75
 CMW50530 Pinus pseudostrobus 2011 GUA
L. jani
 CMW51051 Pinus maximinoi 2010 GUA
 CMW49401 Pinus maximinoi 2010 GUA
 CMW48831 Pinus oocarpa 2010 GUA
 CMW36810 Pinus maximinoi 2010 GUA ∆
 CMW48830 Pinus oocarpa 2010 NIC
 IB35.3c Pinus oocarpa 2010 GUA
85/85
 IB30.2b Pinus oocarpa 2010 GUA
SUBCLADE 1
 CMW51143 Pinus oocarpa 2010 NIC
 IB13.2f Pinus maximinoi 2010 GUA
 N3.2c Pinus oocarpa 2010 NIC
 CMW37128 Pinus oocarpa 2010 GUA
 CMW47109 Pinus maximinoi 2010 GUA
 CMW45388 Pinus maximinoi 2010 GUA
100/94
 CMW36808 Pinus maximinoi 2010 GUA ∆
 CMW45389 Pinus maximinoi 2010 GUA
 267.52.N2S1 Pinus tecunumanii 2012 GUA
 CMW51058 Pinus tecunumanii 2012 GUA
 267.47.N2 Pinus tecunumanii 2012 GUA
 CMW51059 Pinus tecunumanii 2012 GUA
94/95
SUBCLADE 2
 267.52.N1S1 Pinus tecunumanii 2012 GUA
 CMW38958 Pinus oocarpa 2012 GUA T
 267.44.N1 Pinus tecunumanii 2012 GUA
 CMW38950 Pinus oocarpa 2012 GUA
70/*
 267.47.N1 Pinus tecunumanii 2012 GUA
L. acicola
 CMW45428 Pinus palustris 1933 USA
 CMW45427 Pinus strobus 2011 USA T
98/97
 CMW50541 Pinus mugo 2015 LIT
 CMW50542 Pinus mugo 2015 LIT
 CMW45426 Pinus mugo 2009 LIT
99/*
 CMW9985 Pinus radiata 1995 FRA
L. variabilis
 CMW45425 Pinus sp. 2009 MEX
100/100
 CMW45390 Pinus oocarpa 2010 GUA
 CMW42205 Pinus caribaea 1980 HON T
96/93
87/87
 CMW37125 Pinus oocarpa 2010 GUA
 CMW36809 Pinus maximinoi 2010 GUA
 CMW37129 Pinus oocarpa 2010 GUA
L. tecunumanii
 CMW46812 Pinus tecunumanii 2010 GUA
100/83
 CMW46805 Pinus tecunumanii 2010 GUA T
 CMW49403 Pinus tecunumanii 2010 GUA
L. pharomachri
 CMW46810 Pinus oocarpa 2010 HON
100/97
 CMW37132 Pinus tecunumanii 2010 GUA
91/94
 CMW37133 Pinus tecunumanii 2010 GUA
 CMW38974 Pinus oocarpa 2012 GUA
 CMW38947 Pinus oocarpa 2012 GUA
 CMW37136 Pinus tecunumanii 2010 GUA T
 CMW46813 Pinus tecunumanii 2010 GUA
 267.30.N4 Pinus oocarpa 2012 GUA
 CMW37134 Pinus tecunumanii 2010 GUA
100/100
 CMW45392 Pinus oocarpa 2010 GUA
L. guatemalensis
 CMW36812 Pinus maximinoi 2010 GUA
 CMW37122 Pinus oocarpa 2010 GUA
87/84
 CMW51052 Pinus oocarpa 2010 GUA
 CMW42206 Pinus oocarpa 1983 GUA T
100/100
 CMW43895 Pinus oocarpa 2010 GUA
 CMW49402 Pinus oocarpa 2010 GUA
 CMW46817 Pinus oocarpa 2010 GUA
 CMW46811 Pinus oocarpa 2010 GUA
79/70
86/91
 CMW37126 Pinus oocarpa 2010 GUA
86/89
 CMW45387 Pinus oocarpa 2010 NIC
 CMW51142 Pinus oocarpa 2010 NIC
 CMW49400 Pinus oocarpa 2010 NIC
 N3.1c Pinus oocarpa 2010 NIC
L. longispora
100/100
100/100
 CMW45429 L. longispora Pinus sp. 2009 MEX T
L. gloeospora
 CMW45430 L. longispora Pinus sp. 2009 MEX
CMW42645 L. gloeospora Pinus sp. 1983 MEX T
100/100
 CMW45434 Phaeophleospora gregaria
 CMW45435 Phaeophleospora gregaria
100/100
 CMW45432 Phaeophleospora eugeniae
 CMW45433 Phaeophleospora eugeniae
100/100
 CMW44656 Dothistroma septosporum T
 CMW44657 Dothistroma septosporum
 CMW45395 Amycosphaerella africana
100/95
 CMW45396 Amycosphaerella africana
0.05
